# Combining drug salt formation with amorphous solid dispersions – a double edged sword

**DOI:** 10.1016/j.jconrel.2022.09.056

**Published:** 2022-12

**Authors:** Tze Ning Hiew, Lynne S. Taylor

**Affiliations:** Department of Industrial and Physical Pharmacy, College of Pharmacy, Purdue University, West Lafayette, IN 47907, United States

**Keywords:** Lumefantrine, Copovidone, Amorphous solid dispersion, Salt formation, Glass transition temperature

## Abstract

Glass transition temperature (*T*_g_) is important for amorphous compounds because it can have implications on their physical and chemical stability. With drugs that possess ionizable acidic or basic groups, salt formation is a potential strategy to reduce re-crystallization tendency through *T*_g_ elevation. While salt formation has been reported to impact re-crystallization tendency, it is not known if this holds true for all drugs and if it is useful in the context of amorphous solid dispersion (ASD) formulations. In addition, little information on the impact of salt formation on drug release performance of ASD is available. Herein, the influence of salt formation and *T*_g_ elevation on the release performance of lumefantrine (*T*_g_ = 19.7 °C) when formulated as an ASD with copovidone (PVPVA) was examined. Lumefantrine salts and lumefantrine salt–PVPVA ASDs with drug loadings (DLs) ranging from 5 to 30% were prepared. The acids used for salt formation were benzoic acid, benzenesulfonic acid, camphorsulfonic acid, hydrochloric acid, *p*-toluenesulfonic acid, poly(ethylene) glycol 250 diacid (PEG 250 diacid), and sulfuric acid. Salt formation resulted in an elevation of *T*_g_ compared to lumefantrine free base, with the largest increase in *T*_g_ observed with lumefantrine sulfate. With a lower *T*_g_ salt, ASDs could be formulated at higher DLs while ensuring drug release. In contrast, drug release ceased at a DL as low as 5% when *T*_g_ of the salt was high. However, ASDs with lower *T*_g_s such as the benzoate and PEG 250 diacid salts showed poor stability against re-crystallization when stored under stress storage conditions. When using a salt in an ASD formulation, attention should be paid to the *T*_g_ of the salt, since it may show opposing effects on physical stability and drug release, at least for PVPVA-based ASDs.

## Graphical abstract

**Figure:**
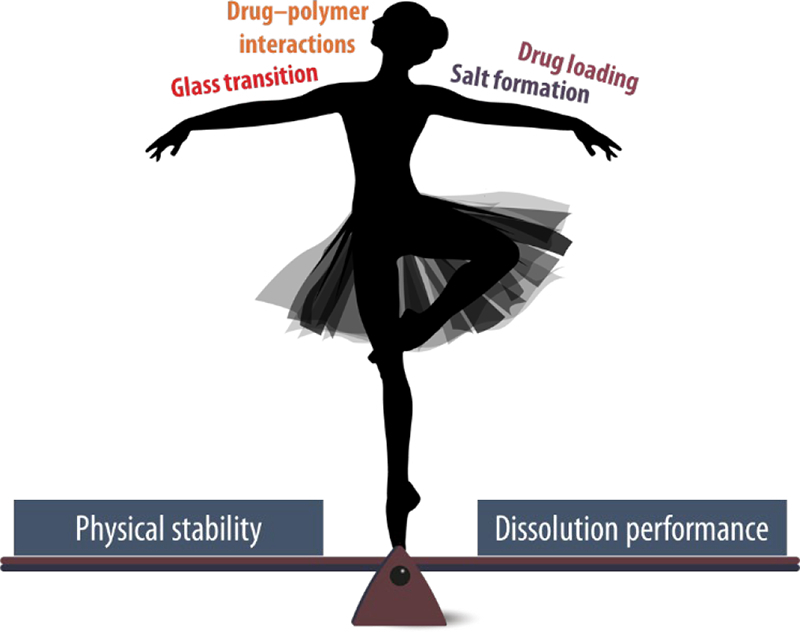

## 1 Introduction

One of the most widespread challenges in the development of oral solid dosage forms is the increased prevalence of molecularly obese lipophilic new chemical entities (NCEs) [1]. Molecular obesity is commonly associated with poor aqueous solubility, which makes these NCEs difficult to administer via the oral route. Different formulation strategies have been leveraged to overcome aqueous solubility limitations including cocrystals, salts, surfactants, cyclodextrins, and amorphous solid dispersions (ASDs) [[2], [3], [4], [5], [6], [7], [8]]. In particular, ASD as an enabling formulation strategy is especially popular due to its supersaturation advantage, which drives rapid and sustained absorption in the gastrointestinal tract following dissolution [9]. Since early 2010, the United States Food and Drug Administration has approved 16 products formulated as ASDs, and 19 products were approved between 2007 and 2017 [2].

An ASD comprises molecularly mixed drug and polymer, where the drug and polymer interact at the molecular level through specific (such as ionic, hydrogen, and halogen bonding) [[10], [11], [12], [13], [14], [15]] and non-specific interactions (van der Waals forces). This formulation approach is desirable when dealing with solubility-limited absorption because the amorphous form of the drug, which has a higher energy, often generates a supersaturated solution upon dissolution, while the polymer is used to kinetically stabilize the supersaturated solution through suppression of nucleation and crystal growth [[16], [17], [18]]. For amorphous compounds, glass transition temperature (*T*_g_) is probably one of the most important solid-state properties, as the physical and chemical stability of these compounds is influenced by the storage temperature relative to the *T*_g_ [19]. The re-crystallization rate of an amorphous compound increases notably above *T*_g_ due to the increased molecular mobility of the supercooled liquid state relative to its glass, as illustrated with amorphous sucrose [20,21]. Therefore, when formulating an ASD, the use of polymers with high *T*_g_s is especially desirable when paired with low *T*_g_ drugs, as the overall *T*_g_ of the resultant dispersion is elevated, [22], decreasing molecular mobility and improving physical stability.

Another factor which affects the stability of an ASD is the presence of specific drug–polymer intermolecular interactions [12,[23], [24], [25], [26]]. Several studies have indicated that drug–polymer hydrogen bonds lead to reduced re-crystallization rates, while a lack of specific interactions may lead to faster re-crystallization. Our previous study with lumefantrine ASDs demonstrated that, in the absence of specific intermolecular interactions with the polymer, the drug rapidly re-crystallized within a short period of time upon exposure to elevated temperature and humidity conditions [10]. In contrast, when ionic interactions were formed with the ASD polymer, the ASDs were physically stable for extended time periods.

Lumefantrine is a weakly basic drug (p*K*_a_ of tertiary amine = 8.73) [27]. Lumefantrine crystals are yellow in color and this compound has not been reported to exhibit polymorphism [28]. The amorphous form has a *T*_g_ of 19.7 °C which, in combination with the absence of an available hydrogen bond donor, may lead to rapid re-crystallization of the drug from ASDs formulated with copovidone (PVPVA) [10]. However, due to its relatively high p*K*_a_, lumefantrine can theoretically form salts with a variety of acids, given that successful salt formation requires a p*K*_a_ difference between acid and base of >2 units [29]. Salt formation is a strategy widely employed to improve the physicochemical properties of ionizable active pharmaceutical ingredients. Advantages associated with salt formation include enhanced dissolution rate, improved solid-state properties, and elevated melting points relative to the free base. For amorphous drugs, salt formation is a potential strategy to reduce re-crystallization tendency through *T*_g_ elevation. For example, Tong and Zografi reported that amorphous Na indomethacin has a *T*_g_ that is ∼75 °C higher than indomethacin free acid [30]. Further, they noted that the ionic radius of the alkali metal cation correlated with the extent of *T*_g_ elevation of the amorphous indomethacin salts [31]. Salt formation also has been shown to impact re-crystallization tendency. For example, the re-crystallization temperatures of amorphous propranolol salts were elevated compared to the free base drug [19]. While these limited studies suggest that salt formation may be a promising strategy to enhance the physical stability of amorphous materials, this topic has been little explored in the context of ASD formulations. Additionally, information is largely absent on the impact of salt formation and subsequent *T*_g_ elevation on drug release performance of ASDs. The increased *T*_g_ via salt formation is a potential concern for drug release because recent studies provided some evidence that systems with high *T*_g_ relative to the dissolution temperature showed impaired release [12,15,32]. This study aims to examine the influence of salt formation on two important ASD metrics, namely the physical stability and release performance of lumefantrine formulated with PVPVA, one of the most commonly used neutral polymers in marketed ASD products [33]. Lumefantrine was reacted with a variety of acids, and the *T*_g_s of the amorphous salts were determined using differential scanning calorimetry. Open dish accelerated stability storage conditions of 40 °C/ 75% relative humidity (RH) were used to store the amorphous salts and ASDs. The samples were monitored for evidence of re-crystallization at fixed timed intervals using powder X-ray diffraction. Release studies were performed using the Wood's intrinsic dissolution apparatus.

## 2 Materials

**Fig. 1:**
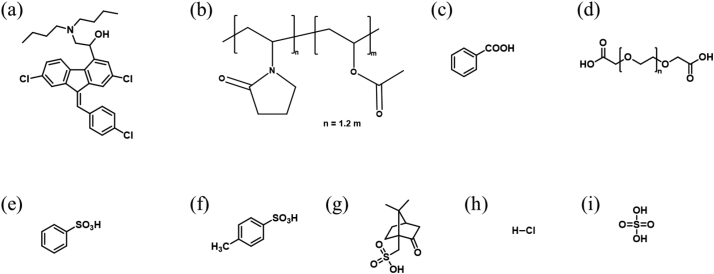
Chemical structures of (a) lumefantrine, (b) PVPVA, (c) benzoic acid, (d) PEG 250 diacid, (e) benzenesulfonic acid, (f) *p*-toluenesulfonic acid, (g) (1*S*)-(+)-10-camphorsulfonic acid, (h) hydrochloric acid, and (i) sulfuric acid.

Lumefantrine (Gojira Fine Chemicals, Bradford Heights, OH) and PVPVA (Kollidon VA 64, BASF Corporation, Ludwigshafen, Germany) were the model drug and polymer used. Dichloromethane (DCM), methanol (MeOH), acetonitrile (MeCN), acetone, tetrahydrofuran, potassium phosphate monobasic, sodium hydroxide, hydrochloric acid (HCl), and sulfuric acid (sulfate) were purchased from Fisher Chemical (Fair Lawn, NJ). Trifluoroacetic acid (TFA) and *p*-toluenesulfonic acid were procured from Acros Organics (Fair Lawn, NJ). Nile red, benzenesulfonic acid (besylate), (1*S*)-(+)-10-camphorsulfonic acid (camsylate), poly(ethylene) glycol 250 diacid (PEG 250 diacid), and benzoic acid (benzoate) were purchased from Sigma-Aldrich (St. Louis, MO). All the dissolution studies were performed in 50 mM phosphate buffer (pH 6.8). Fig. 1 shows the chemical structures of lumefantrine, PVPVA, and the acids used to prepare lumefantrine salts.

## 3 Methods

### 3.1 Preparation of lumefantrine salts and ASDs via solvent evaporation

To prepare the lumefantrine salts, equimolar (drug:acid mole ratio is 1:1) concentrations of lumefantrine and the acid were completely dissolved in a suitable solvent, except for the sulfate salt, where its salt was prepared with half the amount of acid (drug:acid mole ratio is 2:1). For PEG 250 diacid, a salt was also prepared with half the amount of acid (drug:acid mole ratio is 2:1). The solvent system used to prepare each salt is summarized in Table 1. A Hei-VAP Core rotary evaporator (Heidolph Instruments, Schwabach, Germany) coupled to an EcoChyll S cooler (Ecodyst, Apex, NC) was used to remove the solvent at 45 °C. The salts were subsequently dried overnight in a vacuum oven before being used for further analysis.

**Table 1:** Solvent systems used to dissolve various lumefantrine salts.

**Salt**	**Solvent system**
Lumefantrine benzoate	DCM/MeOH 8:2, *v/v*
Lumefantrine PEG 250 diacid	DCM/MeOH 8:2, *v/v*
Lumefantrine besylate	DCM/acetone 8:2, *v/v*
Lumefantrine tosylate	Tetrahydrofuran
Lumefantrine camsylate	DCM/acetone 8:2, *v/v*
Lumefantrine HCl	DCM
Lumefantrine sulfate	DCM/MeOH 8:2, *v/v*

Lumefantrine salt–polymer ASDs were similarly prepared using the solvent systems shown in Table 1. All the samples were prepared at a solids content of 20 mg/mL. The ASDs were further dried overnight in a vacuum oven. A cryogenic impact mill (6775 Freezer/Mill, SPEX SamplePrep, Metuchen, NJ) was used to mill the ASD into powder, and the undersize fraction was collected from a 60 mesh (250 μm) sieve.

### 3.2 Equilibrium solubility measurement

The equilibrium solubility of lumefantrine salts in 50 mM pH 6.8 phosphate buffer at 37 °C was measured by adding an excess amount of salt to 20 mL scintillation vials containing 15 mL of buffer. The suspensions were stirred at 300 rpm for 48 h and carefully decanted into Ultra-Clear centrifuge tubes (Beckman Coulter, Brea, CA), followed by ultracentrifugation at 35,000 rpm for 30 min at 37 °C using an Optima L-100 XP centrifuge (Beckman Coulter, Brea, CA) equipped with a SW 41 Ti swinging-bucket. Lumefantrine concentration in the supernatant was determined via high-performance liquid chromatography (HPLC).

### 3.3 Quantification of lumefantrine

Lumefantrine was quantified with a 1260 Infinity II HPLC system (Agilent Technologies, Santa Clara, CA) with a reversed-phase C18 column (ZORBAX Eclipse Plus, 5 μm particle size, 4.6 mm × 150 mm, Agilent Technologies, Santa Clara, CA) maintained at 25 °C. The mobile phase comprised 70% MeCN and 30% acidified water (0.1% TFA). The isocratic elution mode with a flow rate of 1.5 mL/min was used. For each analysis, the injection volume was 80 μL and lumefantrine was detected at 335 nm using an ultraviolet detector. With these conditions, the retention time of lumefantrine was ∼4.8 min.

### 3.4 Thermal analysis

A Q2000 differential scanning calorimeter (DSC; Q2000, TA Instruments, New Castle, DE) connected to an RCS 90 refrigerated cooling system (TA Instruments, New Castle, DE) was used to analyze the lumefantrine salts and salt ASDs. The thermal properties of lumefantrine free base, lumefantrine free base ASDs, and neat PVPVA were also analyzed. The purging gas used was dry nitrogen (50 mL/min flow rate). Temperature calibration of the DSC was performed using indium and tin, while indium was used for enthalpy calibration. Each sample (5–10 mg) was carefully weighed and sealed in a Tzero aluminum sample pan (TA Instruments, New Castle, DE). The procedure to obtain the sample *T*_g_ is as follows: equilibration at −10 °C, followed by heating with a modulation of ±0.636 °C every 40 s at 3 °C/min to the temperature of interest. The samples were then cooled to −10 °C at 10 °C/min and reheated at 5 °C/min. The first heating cycle was used to remove thermal history associated with the samples, while the second cycle was used to obtain the sample *T*_g_.

### 3.5 Powder X-ray diffraction (PXRD)

PXRD patterns of the lumefantrine salts and salt ASDs were collected using an X-ray diffractometer (Rigaku SmartLab, Rigaku Americas, The Woodlands, TX) operating at 40 kV and 44 mV in Bragg–Brentano mode with a Cu-kα radiation source and D/tex ultra detector. Each sample was spread thinly on a glass sample holder. Data was collected from 4 to 40° 2θ using a step size of 0.02° and a scan rate of 4°/min.

To assess the physical stability of the salts and salt ASDs, the samples were stored in open dish accelerated stability conditions of 40 °C/ 75% RH. At pre-determined time intervals, the samples were removed and their PXRD patterns collected.

### 3.6 Surface normalized dissolution of lumefantrine and lumefantrine salt ASD compacts

The Wood's intrinsic dissolution apparatus (Quality Lab Accessories, Telford, PA) was used to evaluate the surface normalized dissolution of lumefantrine and lumefantrine salt ASD compacts. The compacts were prepared according to the method described previously [10]. A Vision G2 Classic 6 dissolution tester (Teledyne Instruments, Chatsworth, CA) with 250 mL vessels, equipped with a thermostatically controlled water bath set to 37 °C was used to perform the dissolution experiments. For select formulations, dissolution studies were also performed at 10 °C. The dissolution medium used herein was 50 mM pH 6.8 phosphate buffer. At pre-determined time points, aliquots of dissolution medium were withdrawn, diluted, and the drug concentration quantified using HPLC. For selected formulations, PVPVA release during dissolution was also quantified.

### 3.7 Quantification of PVPVA

The amount of PVPVA released during dissolution was quantified according to the method described previously [34,35]. Before quantification, all samples were diluted appropriately with MeCN and ultrapure water (while maintaining an aqueous/organic phase ratio of 1:1) and filtered through a 13 mm diameter 0.45 μm syringe filter (PTFE, Basix, Fisher Scientific, Pittsburg, PA).

### 3.8 Fourier transform infrared (FTIR) spectroscopy

Lumefantrine salts and salt ASDs were dissolved in the solvent systems shown in Table 1 to prepare stock solutions with a solids content of 50 mg/mL. ASD thin films were prepared on zinc selenide windows (Harrick Scientific Corporation, Ossining, NY) via spin coating. To each window, 50 μL of stock solution was carefully deposited with a micropipette and spun for 15 s at 500 rpm, followed by 45 s at 2000 rpm using a KW-4A spin coater (Chemat Technology, Northridge, CA).

A Vertex 70 IR spectrophotometer (Bruker Optics, Billerica, MA) was used to perform FTIR analysis of the ASD thin films, as described previously [10]. For comparison of peak height ratios, baseline correction and normalization were performed on the spectra using the OPUS Software (Version 7.2, Bruker Optics, Billerica, MA).

### 3.9 Scanning electron microscopy (SEM)

#### 3.9.1 Preparation of sample for SEM imaging

The Wood's intrinsic dissolution apparatus was used to prepare ASD compacts for SEM imaging. The compacts were exposed to 50 mM phosphate buffer (pH 6.8) as described in the setup for intrinsic dissolution experiments. At pre-determined time points, the dissolution experiments were halted. The exposed surface of each compact was quickly purged with dry air to remove residual buffer. Once dried, the compacts were carefully ejected from the die and mounted onto aluminum pin stubs for SEM imaging. The compacts were sputter-coated with platinum for 60 s using a 208C High Vacuum Turbo Carbon Coater (Cressington Scientific Instruments, Watford, UK).

#### 3.9.2 SEM imaging

A scanning electron microscope (FEI NOVA NanoSEM, FEI Company, Hillsboro, OR) with an Everhart–Thornley detector was used to image the coated compacts. The compacts were imaged using the following operating conditions: 5 kV accelerating voltage, 3 nm spot size, and ∼10 mm working distance.

### 3.10 Water sorption analysis

Water sorption profiles of the amorphous lumefantrine salts were measured using a DVS Adventure water vapor sorption analyzer (Surface Measurement Systems, Wembley, UK). Powdered salt (5–10 mg) was dried at 0% RH in the instrument for 120 min. After drying, the RH within the chamber was increased from 5% to 95% at a 5% step interval. The equilibrium criterion was a percent weight change over time (% dm/dt) of <0.002%/min for 10 min. Nitrogen was used as the purging gas.

The water uptake of selected lumefantrine salt–PVPVA ASDs at 40 °C and 95% RH was also determined. The sample was equilibrated at 0% RH for 120 min. The RH was then increased to 95% and the sample weight change monitored until the % dm/dt was <0.002%/min for 10 min.

### 3.11 Confocal microscopy

#### 3.11.1 Preparation of stock solutions for spin coating

Double strength ASD stock solutions (200 mg/mL) were prepared in DCM/MeOH 8:2, *v/v*. A 1 mg/mL stock solution was prepared by dissolving Nile red in MeOH. The stock solutions prepared were combined to achieve a final stock solution containing 100 mg/mL ASD and 0.1 mg/mL Nile red and topped up with DCM/MeOH 8:2, *v/v* to final volume before spin coating.

#### 3.11.2 Preparation of ASD films

The stock solutions containing ASD components and Nile red were spin coated on 35 mm glass bottom dishes with a 10 mm micro-well #1.5 cover glass (Cellvis, Mountain View, CA) using a KW-4A spin coater (Chemat Technology, Northridge, CA). For each sample, 30 μL of sample was deposited on the glass before spin coating. All the films prepared were heated at 100 °C for 5 min to ensure film homogeneity and stored under reduced pressure before being used for further analysis

#### 3.11.3 Confocal microscopy imaging of ASD films

A Nikon A1 Confocal and Eclipse Ti2 Inverted Microscope equipped with an Apo 60× oil λS DIC N2 (NA 1.4) objective lens (Nikon, Tokyo, Japan) was used to image the films. The Nile red signal was collected using the 561.5 nm laser line. Confocal images of the compacts were collected using the parameters described previously [35]. ASD films were placed in a stage top incubator (INUBTFP-WSKM-F1, Tokai Hit, Fujinomiya, Japan) set to 37 °C. High RH was generated by evaporation of distilled water in the incubator.

## 4 Results

### 4.1 Equilibrium solubility of lumefantrine salts

The equilibrium solubility of lumefantrine salts in 50 mM pH 6.8 phosphate buffer was lower than the limit of detection of 0.002 μg/mL at 37 °C.

### 4.2 Thermal analysis

**Fig. 2:**
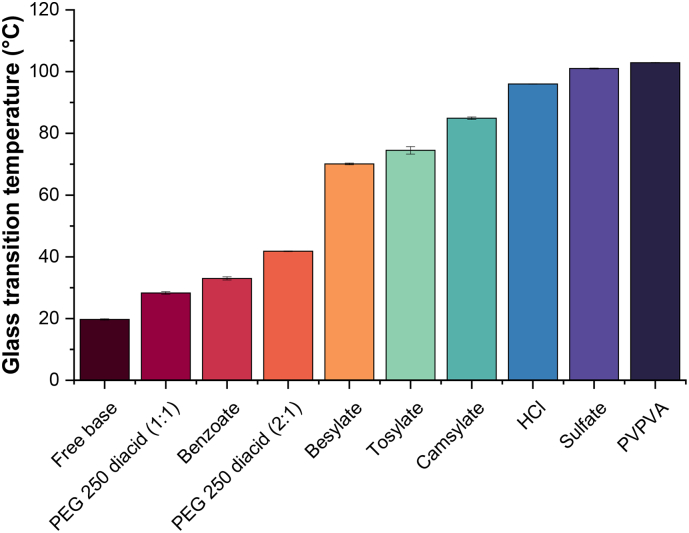
Onset *T*_g_s of lumefantrine free base, salts, and neat PVPVA. Error bars are standard errors of mean, where *n* = 2.

The onset *T*_g_s of lumefantrine free base and its various salts are summarized in Fig. 2. All the samples showed a single glass transition event. Conversion of lumefantrine free base to a salt resulted in an elevation in *T*_g_, with the extent dependent on the counterion. The sulfonate salts had higher *T*_g_s compared to carboxylate salts such as benzoate and PEG 250 diacid salts. Lumefantrine sulfate had the highest *T*_g_, while the benzoate salt only showed a marginal elevation of ∼10 °C in *T*_g_ relative to the free base.

**Fig. 3:**
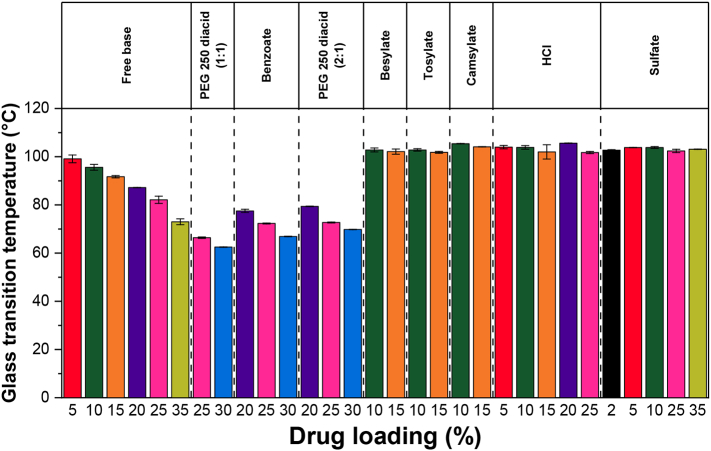
Onset *T*_g_s of lumefantrine free base and salt ASDs prepared with PVPVA. Error bars are standard errors of mean, where *n* = 2.

The onset *T*_g_s of ASDs prepared with PVPVA are shown in Fig. 3. For the free base, benzoate, and PEG 250 diacid ASD formulations, the onset *T*_g_ of the ASDs decreased by ∼5 °C with every 5% increase in drug loading (DL). Little-to-no change in onset *T*_g_ was observed for the besylate, tosylate, camsylate, HCl, and sulfate ASD formulations as DL was increased, and the onset *T*_g_s of these formulations were found to be similar to that of neat PVPVA.

### 4.3 Water sorption profile measurements

**Fig. 4:**
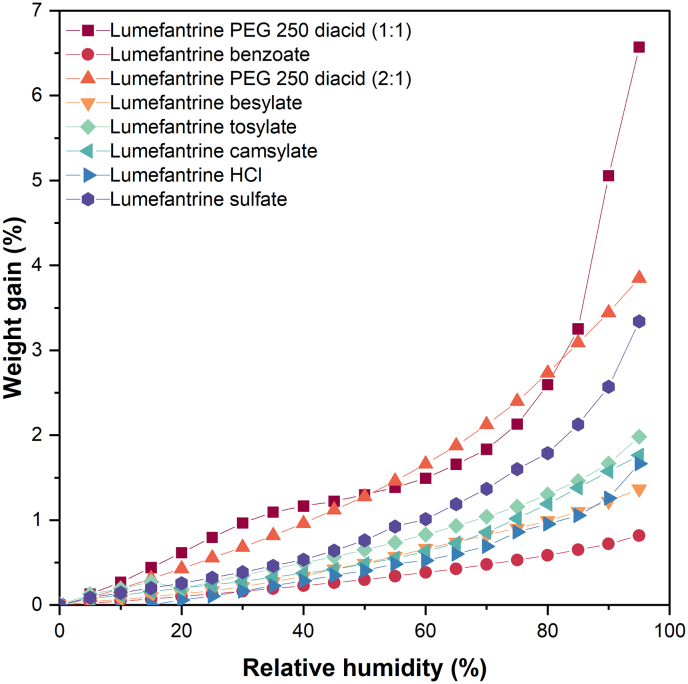
Water sorption profiles of amorphous lumefantrine salts.

Water sorption profiles of the lumefantrine salts are shown in Fig. 4. Among the salts, the PEG 250 diacid salts showed the most weight gain with increasing RH. This was followed by the sulfate salt, which had a 3.3% weight gain at 95% RH. The benzoate, besylate, tosylate, camsylate, and HCl salts had weight gains of <2% at 95% RH. The PEG 250 diacid salt containing excess acid (1:1) was relatively more hygroscopic compared to the other salts. The trend in weight gain for the PEG 250 diacid salts, regardless of the amount of acid added, was similar at lower RHs. However, an upswing in % weight gain was observed for the PEG 250 diacid (1:1) salt at higher RHs; at 90 and 95% RH, this salt showed 5.1 and 6.6% increase in weight, respectively. This salt also liquified, while other salts retained the appearance of a powder after exposure to 95% RH.

### 4.4 Physical stability of lumefantrine salts and ASDs

**Fig. 5:**
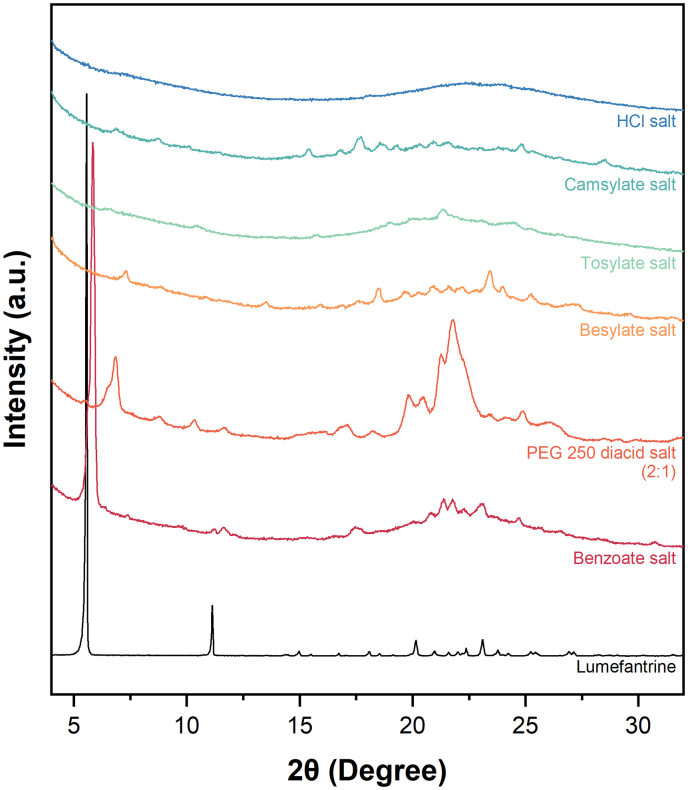
PXRD patterns of re-crystallized lumefantrine salts following open dish storage at accelerated stability conditions of 40 °C/75% RH. The benzoate, PEG 250 diacid (2:1), besylate, tosylate, camsylate, and HCl salts re-crystallized over the course of 32 weeks.

Based on the PXRD patterns of the salts and ASDs, all the freshly prepared salts and dispersions were X-ray amorphous. Among the salts, the benzoate, PEG 250 diacid, and besylate salts had crystalline peaks within 4 weeks of storage at 40 °C/75% RH. Crystalline peaks were observed in the camsylate salt after 32 weeks of storage. The HCl and sulfate salts resisted re-crystallization for at least 16 weeks. The peak positions of the various salts were different from those of lumefantrine free base, which suggests that the re-crystallized salts did not undergo disproportionation during storage (Fig. 5).

**Fig. 6:**
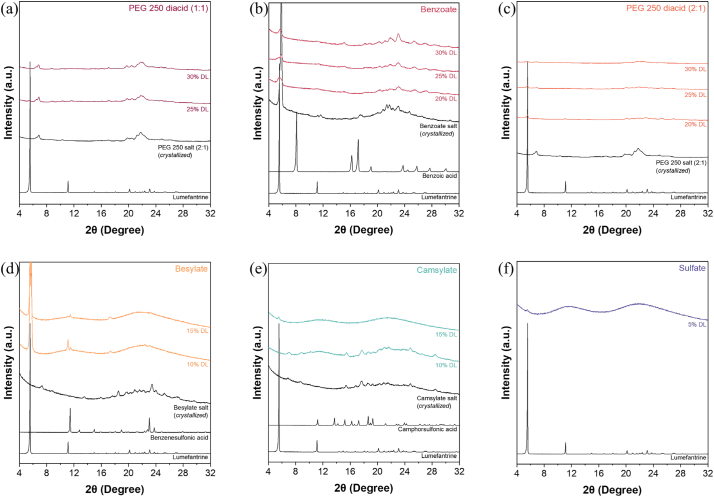
PXRD patterns of various lumefantrine salt ASDs which re-crystallized over time when stored under accelerated stability conditions of 40 °C/75% RH, including (a) lumefantrine PEG 250 diacid (1:1), (b) lumefantrine benzoate, (c) lumefantrine PEG 250 diacid (2:1), (d) lumefantrine besylate, (e) lumefantrine camsylate, and (f) lumefantrine sulfate.

The 20, 25, and 30% DL lumefantrine benzoate–PVPVA and lumefantrine PEG 250 diacid–PVPVA formulations (regardless of the amount of acid added) were found to re-crystallize within a week (Fig. S2a-h). It was observed that with lumefantrine PEG 250 diacid–PVPVA (1:1) ASDs, the drug re-crystallized during storage but did not undergo significant disproportionation, as the peak positions were similar to that of the re-crystallized salt (Fig. 6a). Conversely, disproportionation was observed for the lumefantrine PEG 250 diacid–PVPVA (2:1) ASDs, as a mixture of peaks overlapping with both the lumefantrine free base and PEG 250 diacid salt was observed in the diffractogram (Fig. 6c). With the benzoate salt, the amorphous salt re-crystallized within 4 weeks. Some of the peak positions of the re-crystallized benzoate salt and ASD formulations (Fig. 6b) overlapped with those of lumefantrine free base, which suggests that the salt underwent some extent of disproportionation. Crystalline peaks were observed after 3 days for the lumefantrine besylate–PVPVA 10% DL ASD, while the 15% DL ASD re-crystallized after 2 weeks (Fig. S2i,j). For both the 10 and 15% DL ASDs, the peaks in the re-crystallized samples overlapped with those of lumefantrine free base and benzenesulfonic acid, which suggests that the salts disproportionated when formulated as ASDs (Fig. 6d). Crystalline peaks were also observed after 12 weeks for the lumefantrine camsylate–PVPVA 10% DL ASD, while the 15% DL formulation remained stable for at least 8 weeks, with crystalline peaks observed after 12 weeks (Fig. 6e, Fig. S2k,l). At 10% DL, the camsylate salt ASD appeared to be resistant to disproportionation, as shown by the different peak positions in the re-crystallized salt (Fig. 6e). The lumefantrine sulfate–PVPVA 5% DL ASD remained X-ray amorphous up to 16 weeks (Fig. S2m). The free base–PVPVA ASD at the same DL has been reported to undergo re-crystallization after 8 weeks [10].

### 4.5 Surface normalized dissolution of lumefantrine ASDs

#### 4.5.1 Drug release profiles of lumefantrine ASDs

**Fig. 7:**
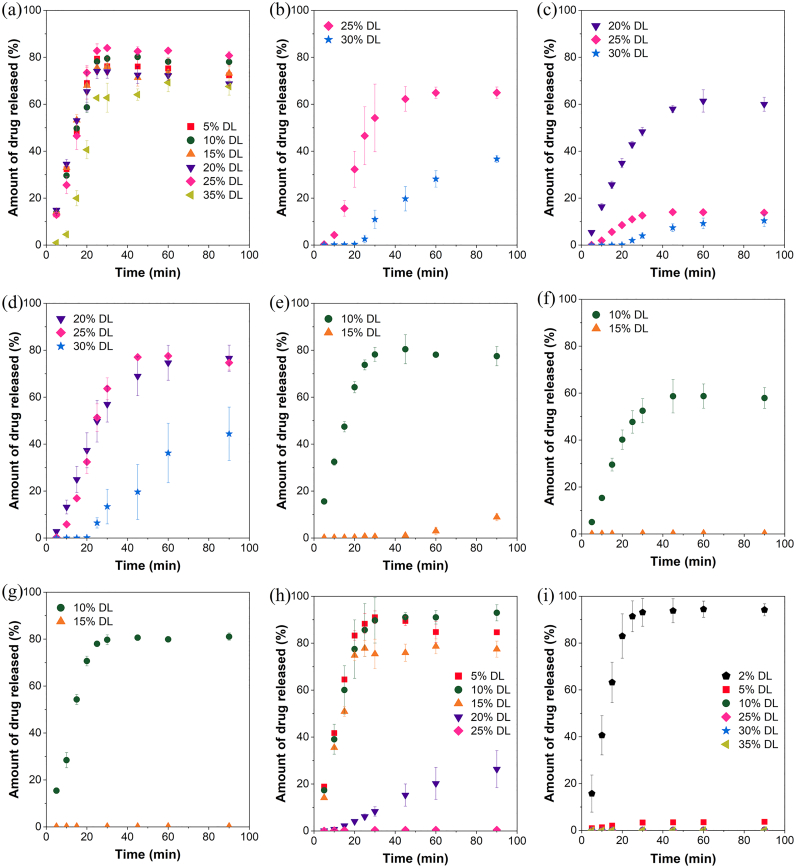
Release profiles of PVPVA-based lumefantrine ASDs prepared with (a) lumefantrine free base, (b) lumefantrine PEG 250 diacid (1:1), (c) lumefantrine benzoate, (d) lumefantrine PEG 250 diacid (2:1), (e) lumefantrine besylate, (f) lumefantrine tosylate, (g) lumefantrine camsylate, (h) lumefantrine HCl, and (i) lumefantrine sulfate. Error bars represent one standard deviation, where *n* = 3.

With the ASD formulations prepared with lumefantrine free base, rapid drug release was observed up to 35% DL (Fig. 7a). Rapid initial drug release was observed for lumefantrine benzoate–PVPVA ASDs for the 20 and 25% DL formulations (Fig. 7c). For the these formulations, a linear increase in drug release with time was initially observed, followed by a plateau at ∼30 min, beyond which no further drug release was observed. Drug release was incomplete for both formulations, where the amount of drug released at 30 min corresponded to ∼60% and ∼15% of the total amount of drug present in the 20% and 25% DL formulations, respectively (Fig. 7c). A lag phase was observed for the 30% DL formulation, where drug release commenced only after 20 min. With the lumefantrine PEG 250 diacid–PVPVA (2:1) ASDs, rapid drug release was observed for the 20 and 25% DL formulations. However, a lag phase was observed for the 30% DL formulations regardless of the drug:acid mole ratio (Fig. 7b,d). Release profiles for the lumefantrine besylate–PVPVA and lumefantrine camsylate–PVPVA ASDs were similar (Fig. 7e,g). The initial drug release was rapid, with near complete release observed for the 10% DL formulations, while little or no release of lumefantrine was detected when the DL was increased to 15%. With lumefantrine tosylate–PVPVA ASDs, rapid drug release was observed initially with the 10% DL formulation, but a plateau was observed after 30 min, even though only ∼60% of the total drug was released (Fig. 7f). With the HCl salt, drug release was rapid for the 5, 10, and 15% DL ASDs, while a decline in the extent and rate of drug release was observed when DL was increased to 20 and 25%, with negligible drug release observed for the 25% DL ASD (Fig. 7h). When formulated as a sulfate salt, rapid and near complete drug release was observed for the 2% DL ASD (Fig. 7i). At 5% DL, the amount of drug release after 90 min of dissolution was ∼0.1 mg, and the amount of drug released with higher DL formulations was even lower (Fig. S3i).

For some ASDs, even though rapid drug release was observed initially, a plateau was observed at ∼30 min. This observation could be attributed to water-induced phase separation as dissolution progressed, which resulted in the formation of a drug-rich layer on the compact surface, thereby preventing further drug release [10,13,36]. Water-induced phase separation and the consequent formation of a drug-rich barrier is often observed with higher DL ASDs.

**Fig. 8:**
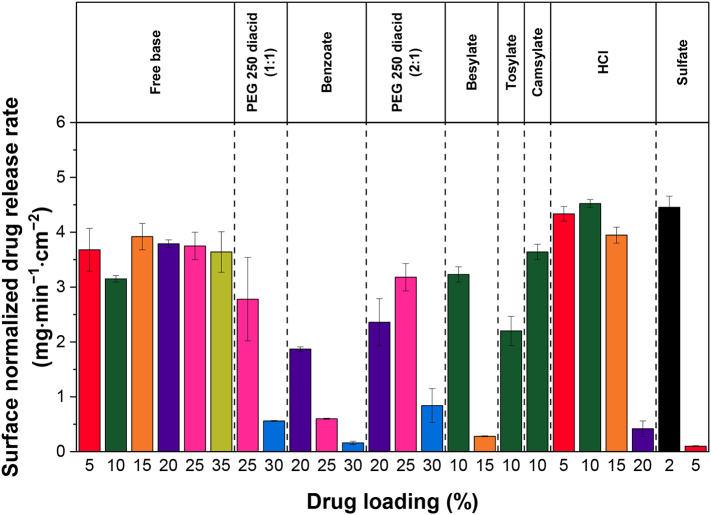
Surface normalized drug release rates of lumefantrine free base and salt ASDs. Error bars represent one standard deviation, where *n* = 3.

The surface normalized drug release rates were calculated from the linear portion of the drug concentration versus time profiles (Fig. 8). All the formulations with near complete drug release had normalized release rates higher than 3 mg·min^−1^·cm^−2^.

#### 4.5.2 Polymer release profiles of lumefantrine ASDs

**Fig. 9:**
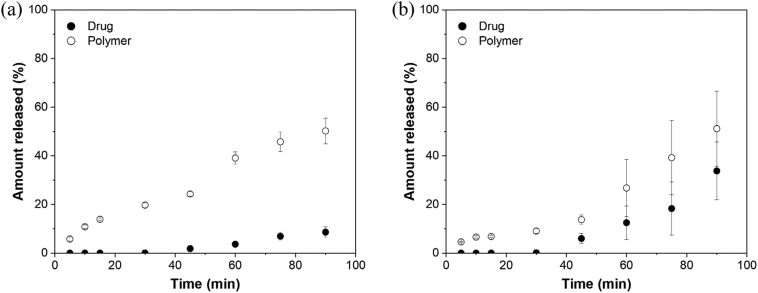
The amount of drug and polymer released from (a) lumefantrine benzoate–PVPVA and (b) lumefantrine PEG 250 diacid–PVPVA 30% DL ASDs. Error bars reflect one standard deviation, where *n* = 3.

To further investigate the release profiles of formulations exhibiting a lag phase, the amount of polymer released over time was quantified for the 30% DL lumefantrine benzoate–PVPVA and lumefantrine PEG 250 diacid–PVPVA (2:1) ASD formulations. Drug and polymer release over a 90 min period is summarized in Fig. 9. While minimal lumefantrine was released during the initial 30 min of dissolution, ∼20% of PVPVA was released (Fig. 9a). PVPVA release from the 30% DL lumefantrine benzoate–PVPVA ASD showed a biphasic profile. The release of PVPVA approached a plateau during the first 45 min, followed by a further increase in the amount of polymer released. Interestingly, this increase corresponded to the onset of some lumefantrine release. However, the release of PVPVA was consistently faster than lumefantrine, indicative of incongruent release behavior. After 90 min, neither the drug nor polymer achieved complete release, with only ∼10% and ∼50% release observed for the drug and the polymer, respectively. It was interesting to note that lumefantrine release only commenced after ∼20% of PVPVA was released, and the DL was effectively increased to ∼57% following partial PVPVA release. As some lumefantrine was released into the dissolution medium, additional PVPVA was released, with the amount of PVPVA released consistently higher than that of lumefantrine. The disparity in the amount of drug and polymer released over time could account for the low overall amount of drug entering the solution phase (∼10%) because the drug and the polymer were releasing incongruently. Eventually, the release of both the drug and the polymer reached a plateau.

With PEG 250 diacid–PVPVA (2:1) 30% DL ASD, PVPVA release from lumefantrine was slightly slower, with ∼10% of polymer released after 30 min. After 30 min, drug and polymer release started increasing linearly, with the amount of polymer released consistently higher than that of the drug by ∼20% (Fig. 9b). Taking into account the ∼10% of polymer released during the initial 30 min, the effective DL of the formulation was increased from 30% to ∼33% when drug release commenced, which is a more marginal change compared to the lumefantrine benzoate–PVPVA formulation. This could explain why lumefantrine and PVPVA continued to release linearly over the subsequent 60 min dissolution period.

#### 4.5.3 Surface normalized dissolution at lower temperature

**Fig. 10:**
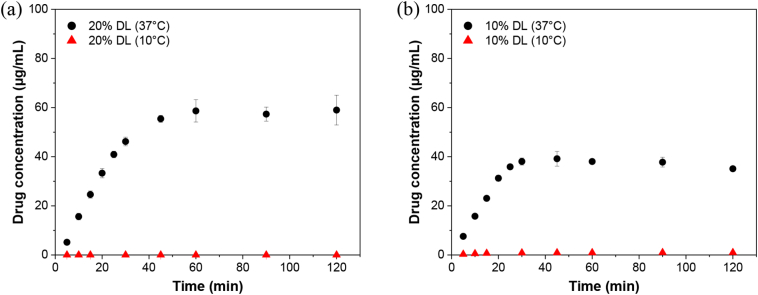
Drug release from (a) lumefantrine benzoate–PVPVA 20% DL and (b) lumefantrine besylate–PVPVA 10% DL ASDs at 10 and 37 °C. Error bars reflect one standard deviation, where *n* = 3.

To better understand the impact of *T*_g_ relative to the dissolution temperature on ASD release performance, dissolution experiments were performed at 10 °C. Fig. 10 shows the drug release profiles of 20% DL lumefantrine benzoate–PVPVA and 10% DL lumefantrine besylate–PVPVA ASDs at 10 and 37 °C. The amount of drug released at 37 °C corresponded to ∼60% and ∼80% of the total drug for the 20% DL lumefantrine benzoate–PVPVA and 10% DL lumefantrine besylate–PVPVA ASDs, respectively. Drug release was dramatically reduced for both systems at 10 °C. This shows that the dissolution temperature relative to *T*_g_ appears to play a role in release performance of ASDs, which is consistent with other studies [12,15].

### 4.6 Scanning electron microscopy (SEM)

**Fig. 11:**
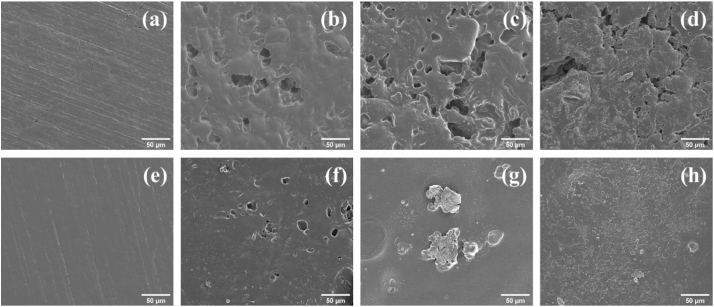
SEM images of lumefantrine benzoate–PVPVA 30% DL compacts taken after (a) 0 min, (b) 10 min, (c) 60 min, and (d) 90 min dissolution, as well as SEM images of lumefantrine PEG 250 diacid–PVPVA (2:1) 30% DL compacts taken after (e) 0 min, (f) 10 min, (g) 60 min, and (h) 90 min dissolution. All the images were taken at 1000× magnification.

The surfaces of partially dissolved compacts of selected ASDs were imaged using SEM to better understand the changes in the solid-solution interface and how it could potentially affect drug and polymer release. For the lumefantrine benzoate–PVPVA 30% DL ASD, large pits were observed across the compact surface after 10 min of dissolution (Fig. 11b). The surface of the compacts also became very rough and irregular. The pits are postulated to have formed as a result of phase separation to a discrete PVPVA-rich phase followed by preferential PVPVA release, as no drug release was detected. Similar surface morphology was observed at the 30 and 60 min time points, with more pits formed at 60 min (Fig. 11b,c). Interestingly, the appearance of the agglomerated structures on the compact surface coincided with increased lumefantrine release (Fig. 9a), which began after approximately 45 min of immersion in the dissolution medium.

The surface of the lumefantrine PEG 250 diacid–PVPVA 30% DL ASD compact was relatively smoother, with 10–30 μm diameter pits observed across the compact surface (Fig. 11f). These pits are also postulated to have formed due to preferential PVPVA release. As dissolution progressed, the surface of the compacts appeared to become less pitted (Fig. 11g,h).

### 4.7 FTIR analysis

**Fig. 12:**
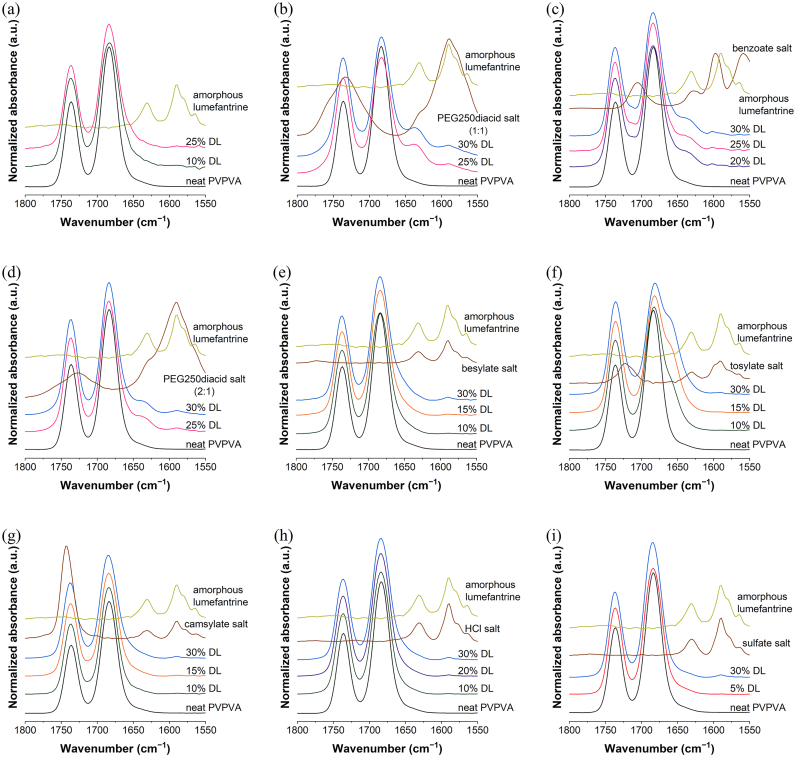
Partial FTIR spectra of PVPVA-based ASDs of (a) lumefantrine free base, (b) lumefantrine PEG 250 diacid (1:1), (c) lumefantrine benzoate, (d) lumefantrine PEG 250 diacid (2:1), (e) lumefantrine besylate, (f) lumefantrine tosylate, (g) lumefantrine camsylate, (h) lumefantrine HCl, and (i) lumefantrine sulfate. Neat PVPVA and amorphous lumefantrine spectra are shown for comparison.

FTIR spectroscopy was used to probe for hydrogen bond formation between the lumefantrine salts and the carbonyl region of PVPVA. The peak located at 1736 cm^−1^ corresponds to the polymer vinyl acetate group, while the peak located at 1684 cm^−1^ corresponds to the polymer vinylpyrrolidone group. With the exception of lumefantrine tosylate–PVPVA ASDs, no peak shift was observed in either peak in the carbonyl region of PVPVA regardless of DL (Fig. 12). However, changes in peak height ratios were observed for several ASDs as DL was increased (Fig. S4). This suggests that for some of the ASDs, there might be a minor extent of intermolecular interactions between lumefantrine salts and the carbonyl groups of PVPVA; these changes were more obvious for the non-stoichiometric salt with excess acid groups (Fig. 12b).

## 5 Discussion

### 5.1 The advantage of the polymer-controlled dissolution regime in PVPVA-based ASDs

Recent studies with PVPVA-based ASDs have shown that for fast releasing ASDs, the drug and the polymer release at the same normalized rate (i.e., congruently). Compared to the release of neat amorphous lipophilic drug, the release rate of the neat polymer is much faster. Thus, in the polymer-controlled regime, the drug release rate is also enhanced, as observed for several PVPVA-based systems studied [10,12,13,15,32,36]. Consequently, release from ASDs should ideally be polymer-controlled to maximize the effectiveness of an ASD formulation. For DLs below a certain value, PVPVA-based ASDs display a consistent release pattern as the DL is increased, where the drug releases at approximately the same normalized rate as the neat polymer. However, once a critical DL is reached that is specific to each drug–PVPVA ASD, the drug release rate falls drastically, in what is known as the “falling-off-a-cliff” phenomenon [36]. This observation has been attributed to a barrier drug-rich layer formed on the compact surface once the DL exceeds a particular value, hence release now becomes drug-controlled, accounting for the observed decrease in the rate of drug release and in some instances, polymer release [10,36]. Amorphous–amorphous phase separation [37], where the intermolecular interactions between the amorphous drug and polymer are disrupted and they are no longer intimately mixed, is thought to contribute to the formation of the contiguous drug-rich barrier layer for higher DL ASDs with poor release. The DL where drug release falls off a cliff varies for different drugs, and the highest DL where congruency is observed is the limit of congruency (LoC). To date, studies with different PVPVA-based systems have reported LoC values ranging from 5 to 35%, but the factors causing drug release to fall off a cliff remain poorly understood [10,12,13,15,32,36]. Some of the factors which have been suggested to promote drug release at higher DLs include a lack of specific drug–polymer interactions and lower drug *T*_g_s [10,15]. In contrast, drugs with a high *T*_*g*_ appear to show low LoCs when formulated with PVPVA, although the role of *T*_*g*_ is not well understood.

### 5.2 Role of *T*_g_ on ASD release

**Fig. 13:**
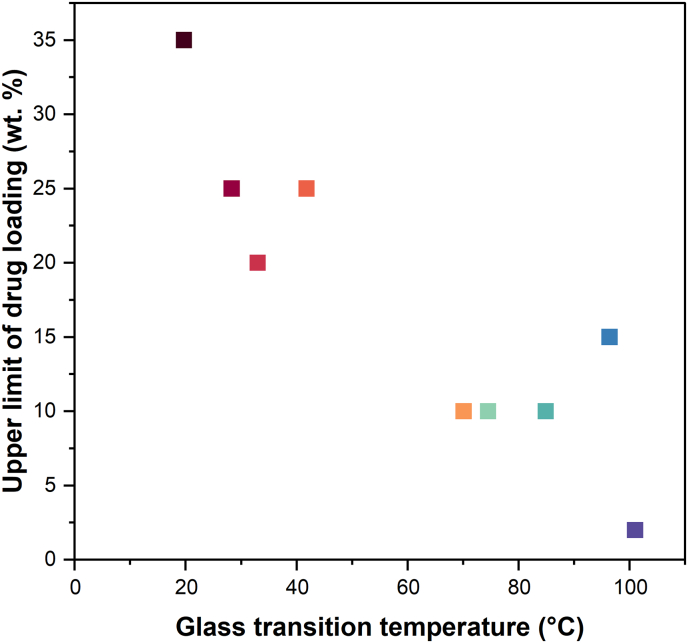
The upper limit of DL where good release was observed reduced as *T*_g_ of the neat salt increased.

One challenge in understanding how *T*_g_ affects drug release from ASDs is to deconvolute the *T*_g_ effect from the impact of specific drug–polymer interactions. This is because drugs with higher *T*_g_s tend to have hydrogen bonding groups and thus form specific interactions with PVPVA, while many drugs with low *T*_g_s lack hydrogen bonding interactions. In a study comparing indomethacin and indomethacin ester, Saboo et al. reported that indomethacin, which hydrogen bonds extensively with PVPVA, had an LoC of 10%, while indomethacin ester, which does not form hydrogen bonds with PVPVA, had an LoC of 25% [12]. However, indomethacin also has a higher *T*_g_ compared to indomethacin ester. In another study comparing several compounds with different *T*_g_s, the high *T*_g_ compounds had hydrogen bond donors, whereas the low *T*_g_ compounds did not; correspondingly, LoC DLs were much greater for ASDs formulated with the non-interacting low *T*_g_ compounds [15]. With lumefantrine and its salts, FTIR spectra (Fig. 12) of the ASDs indicated that the tendency of the salt to interact with PVPVA was in general, very low. Only the PEG 250 diacid/sulfate salts with an unreacted acid group showed some indication of drug–polymer hydrogen bonding. Thus, the specific interactions between stoichiometric lumefantrine salts and PVPVA appear to be less extensive than other systems studied previously [12,15,38]. The sulfate salt (2 mol lumefantrine: 1 mol sulfate) ASD provides an interesting contrast to the lumefantrine free base ASD since neither of these systems show compelling evidence of specific drug–polymer interactions. The LoC for the sulfate salt ASD is 2% and that of the free base is 35%, while the corresponding drug *T*_g_ values are 101 °C and 20 °C, respectively. Based on this analysis, *T*_g_ appears to play an important role in impacting release. This is further illustrated by Fig. 13, which shows how the LoC trends with the drug *T*_g_. It can be clearly seen that the upper limit DL where there is rapid release of the drug and similar to that of the polymer (Fig. 8) declines precipitously as the *T*_g_ increases. This observation suggests that the drug *T*_g_ may be an important factor impacting the achievable DL limit for PVPVA ASDs, even for an ASD without drug–polymer specific interactions.

### 5.3 Implications of drug *T*_g_ variation on the physical stability of ASDs

Low drug *T*_g_ is typically a concern for ASD physical stability. Herein, we note that ASDs with the free base drug and salts with lower *T*_g_ such as the benzoate and PEG 250 diacid salts showed poor stability against re-crystallization when stored under stress storage conditions. Lumefantrine is lipophilic (log *P* = 8.34) [39] while PVPVA is hydrophilic and hygroscopic. When exposed to high humidity conditions, the absence of specific drug–polymer interactions and high drug lipophilicity predisposed the free base–PVPVA system to water-induced phase separation (Fig. S6–8) [[40], [41], [42]]. The benzoate salt ASD was also observed to undergo water-induced phase separation, while the sulfate salt remained visually homogeneous.

Once phase separated, the low *T*_g_s of lumefantrine and some of its salts further increased the risk of drug re-crystallization as a consequence of the lack of protection from the polymer. ASD formulations with relatively higher DLs and low *T*_g_ drug salts were especially susceptible to re-crystallization when exposed to high temperature and humidity, with crystalline peaks appearing as soon as 3 days. The drug/drug salt *T*_g_ thus appears to be an important predictor of the physical stability of the ASD formulation, especially as DL is increased, exacerbated by the tendency of the system to undergo amorphous–amorphous phase separation. For example, a 10% DL PVPVA-based ASD with lumefantrine free base re-crystallized within 2 weeks [10], whereas converting the free base drug to its camsylate salt delayed re-crystallization by ∼10 weeks at the same DL. With the sulfate salt, crystalline peaks were observed for the 5% DL formulation after 12 weeks, while re-crystallization was observed at 4 weeks for the free base formulation at the same DL.

### 5.4 Release mechanism of PVPVA

Since polymer plays such an important role in the overall drug and polymer release in ASDs, especially when release is polymer-controlled, the mechanisms by which PVPVA dissolves should also be taken into consideration, as the critical rate processes for dissolution differ between polymers and small molecules. Studies have shown that polymer dissolution is controlled by three transport processes, water diffusion into the matrix, chain disentanglement from the plasticized gel, and chain diffusion in the boundary layer, and these transport processes can be further modeled to predict polymer dissolution behavior upon contact with a miscible solvent. However, the prediction and modeling of polymer dissolution is often challenging because of diffusion anomalies, i.e., deviation from classical Fickian diffusion, in particular for initially glassy polymers which undergo a glass transition following solvent penetration [43]. Therefore, separate models have been developed for the dissolution of glassy and rubbery polymers [43,44]. For polymers with *T*_g_ higher than the experimental temperature, a critical polymer volume fraction (*v*_p_^∗^) was introduced to account for the amount of solvent required to plasticize the polymer before chain disentanglement can occur [45]. PVPVA, with a *T*_g_ of 102.9 °C, is an example of a glassy polymer with respect to the dissolution temperature of 37 °C. To calculate the critical polymer volume fraction of PVPVA, the following Eq. was used:

(1) $$v_{p}^{*}=\frac{\frac{1}{\rho_{p}}}{\frac{1}{\rho_{p}}+\frac{\left(T_{g}-T\right)}{\left(\beta /\alpha_{f}\right)}∙\frac{1}{\rho_{s}}}$$

Here, *ρ*_*p*_ and *ρ*_*s*_ are the polymer and solvent densities, respectively, *T*_g_ is the glass transition temperature, *T* is the experimental temperature, *β* is the expansion coefficient contribution of the solvent to the polymer, and *α*_*f*_ is the difference between the linear expansion coefficient of the polymer above and below its *T*_g_. The *β*/*α*_*f*_ term was calculated based on the method proposed by Fujita and Kishimoto [46].

The solvent volume fraction, in turn, affects the polymer disentanglement rate (*k*_d_), as demonstrated by the following Eq. [43]:

(2) $$k_{d}=\frac{B}{{\left(1-v_{p}\right)}^{1.9}}$$

where *B* is a constant related to the polymer molecular weight, solvent viscosity, and temperature, while *v*_p_ is the volume fraction of the solvent in the polymer. Eq. (2) shows that above *v*_p_^∗^, the polymer disentanglement rate is affected by the extent of hydration.

Eq. (1) shows that *T*_g_ is important for polymer mobility and relaxation. A larger differential between *T* and *T*_g_ is expected to translate into more solvent required to plasticize the polymer, without which polymer relaxation and disentanglement will be slow, ultimately causing a decrease in dissolution rate. This was shown to be the case for a number of polymers [45]. With a true density value of 1.19 g/cm^3^ [47], the critical polymer volume fraction of PVPVA is estimated to be 0.89, which means that at least 11% water is required to initiate polymer chain disentanglement at 37 °C. However, even if the polymer chains start to disentangle, more water is required to sufficiently reduce the viscosity at the water–polymer interface for the polymer to start dissolving. Water vapor sorption studies showed that PVPVA contained ∼45% moisture at 95% RH, which is considerably more than the theoretical amount required to plasticize the polymer (*v*_p_ > *v*_p_^∗^), and PVPVA dissolution at 37 °C was shown to be rapid and complete [10]. However, the impact of drug additives and the resultant changes in overall *T*_g_ and polymer disentanglement kinetics in terms of impact on the dissolution rate of PVPVA-based ASDs has not been evaluated and requires further consideration.

### 5.5 Impact of *T*_g_ on drug release in ASDs

In general, the LoCs of the lumefantrine ASDs were observed to scale with the *T*_g_ of the lumefantrine salt, where lower LoCs were observed for salts with higher *T*_g_s. For ASDs, the drug can be considered an additive to the polymer, which changes the overall *T*_g_ of the ASD. As lumefantrine and its salts have a lower *T*_g_ compared to PVPVA, the resultant ASDs have lower *T*_g_s compared to neat PVPVA (Fig. 3). By extension, the amount of solvent required to plasticize the ASD before chain disentanglement commences should be lower, which should translate into a faster dissolution rate. However, this may be balanced by a reduced extent of water uptake due to the presence of the lipophilic drug. PVPVA is more hygroscopic compared to lumefantrine, and PVPVA-based lumefantrine ASDs acquired between 35 and 55% of water at 95% RH (Table S1). The neat amorphous lumefantrine salts, with the exception of the PEG 250 diacid and sulfate salts, did not take up >2% of water at 95% RH (Fig. 4). Water vapor sorption studies further showed that formulations containing more PVPVA (i.e., lower DL) had higher moisture uptake. If the *T*_g_ is reduced by 10 °C for every 1% *w/w* of water absorbed [48], the wet *T*_g_ of all the ASDs should be lower than the dissolution temperature of 37 °C. Therefore, it is difficult to rationalize the observed release behavior solely by considering the wet *T*_g_ of the ASDs, and the disentanglement model presented in Eq. (1).

One potentially relevant observation is that the maximum achievable normalized drug release rate (for releasing formulations) was similar to that of neat PVPVA. For polymer to dissolve from the ASD, as well as for chain disentanglement, as discussed above, solvation of the polymer chain is required. Polymer solvation requires disruption of any drug–polymer interactions by the solvent. This should be facile for lumefantrine ASDs due to the low extent of drug–polymer specific interactions. Further, if drug–polymer phase separation occurs in the gel layer formed at the interface of the solid with the aqueous medium, then the normalized release rate of both components would be anticipated to be similar to the normalized release rate of the neat polymer, as long as the phase-separated drug is unable to form a continuous barrier layer. However, separation of polymer and drug species requires molecular mobility. There is an asymmetry in the *T*_g_s of the hydrated polymer and the hydrated drug salt, for the high *T*_g_ drug salts, because the neat amorphous drug salts absorb considerably less water than the neat amorphous polymer, hence will be plasticized to a much lower extent. The concept of a phase separation process that is thermodynamically favorable but is arrested by asymmetry in the dynamics of the two phases is well established for polymer solvent blends, as well other systems [49,50]. This potential explanation is supported by two experimental observations. First, we note that ASDs that released well at 37 °C became poorly releasing systems when the dissolution temperature was decreased (Fig. 10). Second, lumefantrine ASDs with low *T*_g_ salts were observed to undergo water-induced phase separation (free base and benzoate salt ASDs, Figs. S6 and S7), while no obvious phase separation could be detected in an ASD of a high *T*_g_ salt (sulfate salt, Fig. S8).

With salt formation, different counterions resulted in different extents of *T*_g_ elevation. Because of the increase in *T*_g_, any phase separation that occurs following exposure to water is expected to become increasingly detrimental to release due to the corresponding increase in the *T*_g_ of the drug-enriched phase, as a larger *T*_g_ – *T* translates into lower *v*_p_^∗^ and more solvent required to plasticize the system. In the case of a high *T*_g_ compound such as lumefantrine sulfate, the 5% DL ASD with PVPVA was found to be physically stable, but negligible drug release was observed.

### 5.6 Implications for ASD formulation

With drugs that possess ionizable acidic or basic groups, salt formation can be used to modify their solid-state properties and behavior in solution, including solubility, dissolution rate, supersaturation, and bioavailability [51]. Salt formation can also be a strategy to reduce re-crystallization tendency through *T*_g_ elevation [19]. With the same drug, salt formation with different counterions was shown to produce salts with different *T*_g_s. Herein, it was shown that *T*_g_ elevation, while improving the physical stability of the ASD, resulted in a decrease in the LoC. This potentially limits the DL of an ASD formulation where rapid release is observed. Therefore, when using a salt in an ASD formulation, attention should be paid to the *T*_g_ of the salt, since it may show opposing effects on physical stability and drug release, at least for PVPVA-based ASDs.

## 6 Conclusion

Salt formation and *T*_g_ elevation were found to affect the physical stability and dissolution performance of lumefantrine–PVPVA ASDs. With a lower drug *T*_g_, ASDs could be formulated with higher DLs while ensuring drug release. However, these formulations had a higher propensity to re-crystallize. In contrast, drug release ceased at a DL as low as 5% when *T*_g_ of the salt was high, even though salts with higher *T*_g_ and their ASDs were more resistant to re-crystallization. This finding was enabled by utilizing a low *T*_g_, weakly basic drug, lumefantrine to form salts with a series of acidic counterions to elevate the salt *T*_g_ to different extents. It appears that there is a trade-off between improving ASD physical stability through *T*_g_ elevation and maintaining good drug release at higher DLs. This study highlights the role of drug *T*_g_ in the release behavior of ASDs formulated with PVPVA.

## CRediT authorship contribution statement

**Tze Ning Hiew:** Conceptualization, Methodology, Formal analysis, Investigation, Data curation, Writing – original draft, Visualization.

**Lynne S. Taylor:** Conceptualization, Writing – review & editing, Supervision, Funding acquisition.

## Data Availability

No data was used for the research described in the article.

## Appendix A Supplementary data

Supplementary material. Physical stability of lumefantrine salts, physical stability of lumefantrine amorphous solid dispersions (ASDs), surface normalized dissolution of lumefantrine ASDs, weight change of lumefantrine salt ASDs, FTIR analysis, subtraction spectroscopy, confocal microscopy. Image 1
