# Supplementary material for: Combining drug salt formation with amorphous solid dispersions – a double edged sword
Source: J Control Release. 2022 Dec;352:47–60. doi: 10.1016/j.jconrel.2022.09.056 (PMC9733678; doi:10.1016/j.jconrel.2022.09.056)
Supplement: Supplementary file 1 — Supplementary material. Physical stability of lumefantrine salts, physical stability of lumefantrine amorphous solid dispersions (ASDs), surface normalized dissolution of lumefantrine ASDs, weight change of lumefantrine salt ASDs, FTIR analysis, subtraction spectroscopy, confocal microscopy. [file mmc1.docx]

# Supporting information for the manuscript entitled

**Combining drug salt formation with amorphous solid dispersions – a double edged sword**

Tze Ning Hiew, Lynne S. Taylor*

Department of Industrial and Physical Pharmacy, College of Pharmacy, Purdue University, West Lafayette, Indiana 47907, United States

*Corresponding author: Department of Industrial and Physical Pharmacy, Purdue University, 575 Stadium Mall Drive, West Lafayette, Indiana 47907, USA.

## Physical Stability of Lumefantrine Salts

| 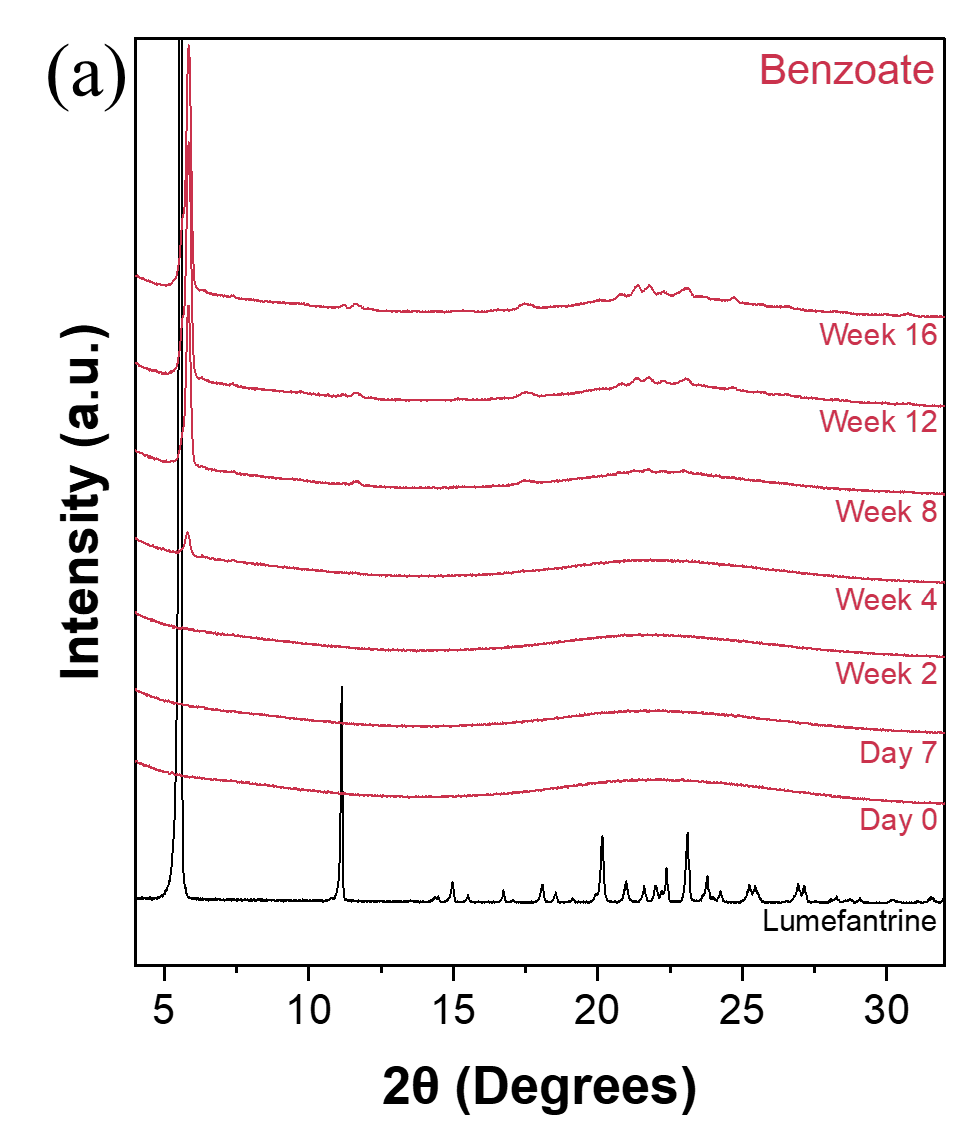 | 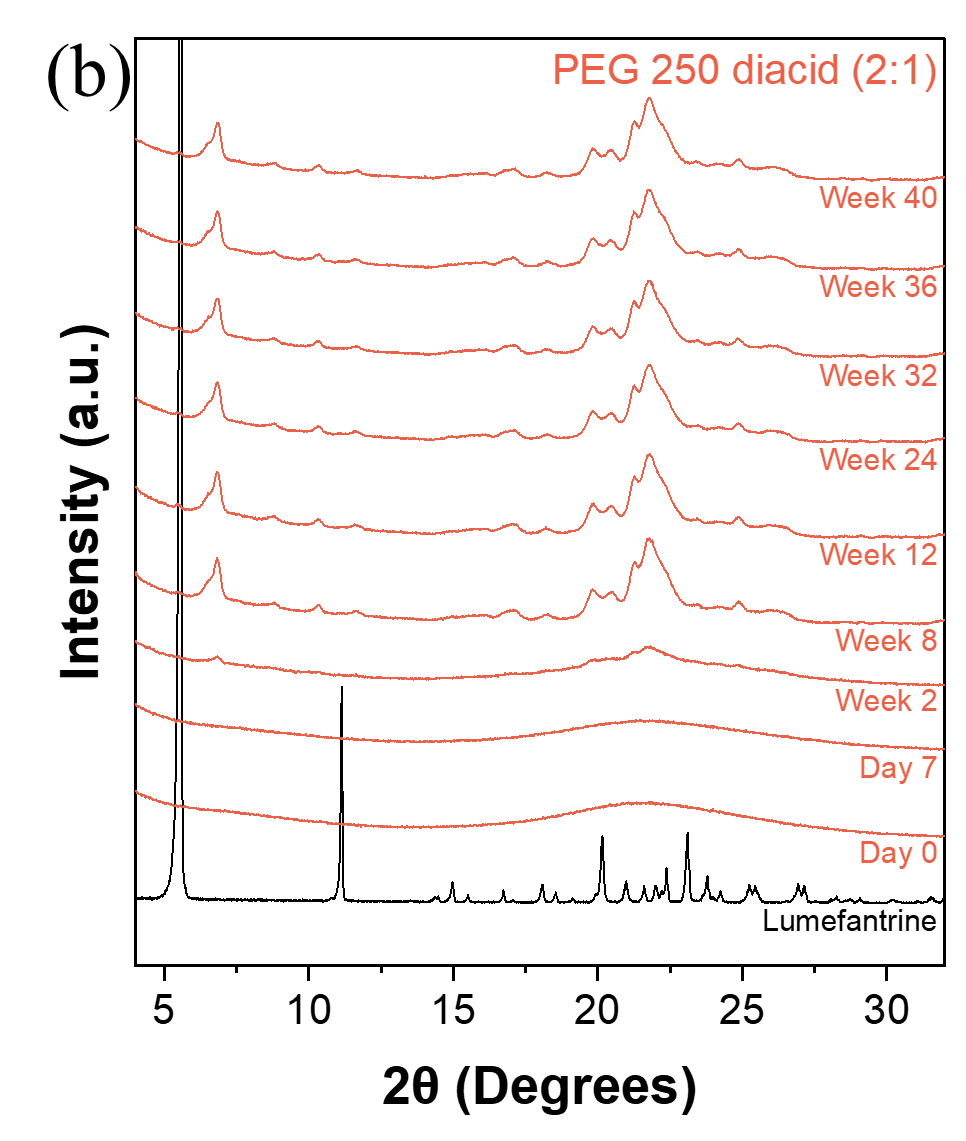 | 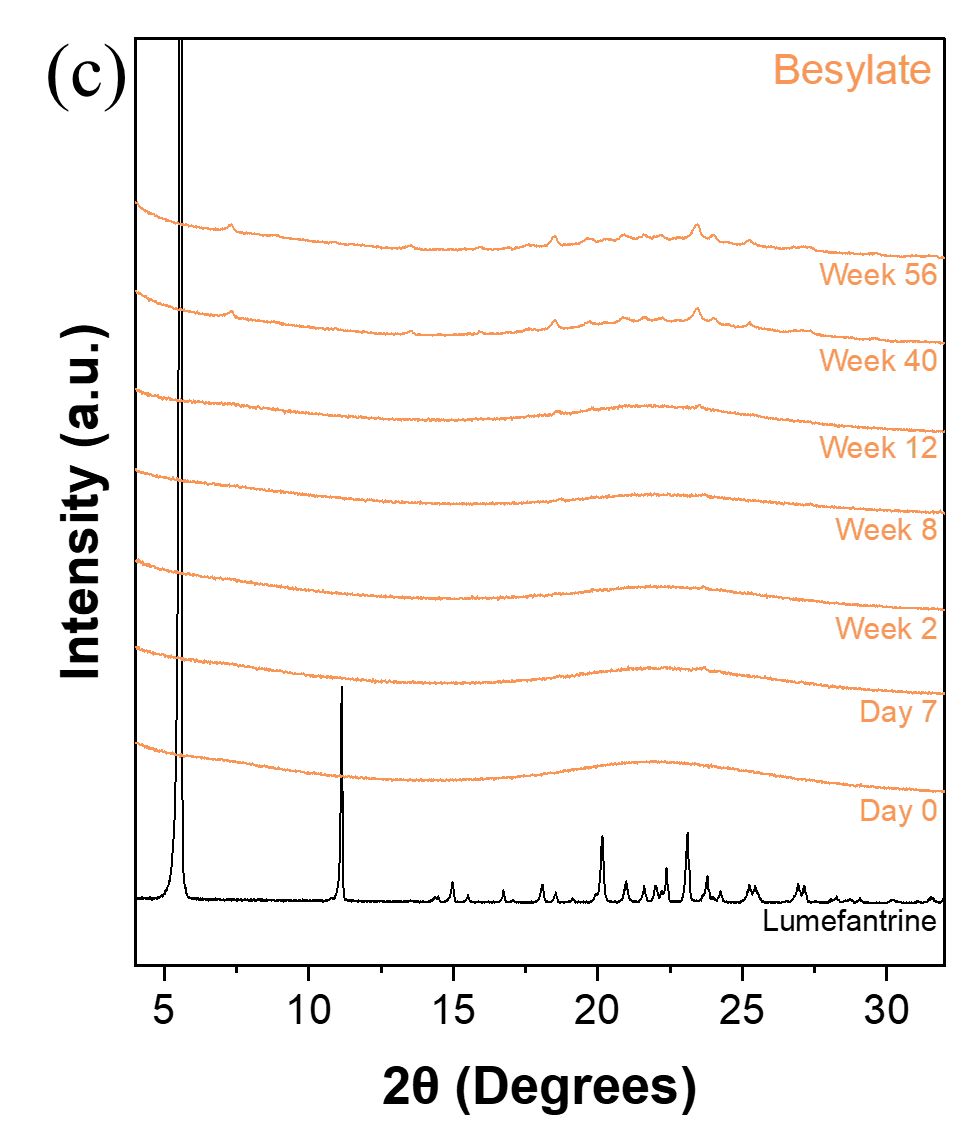 |
| --- | --- | --- |
| 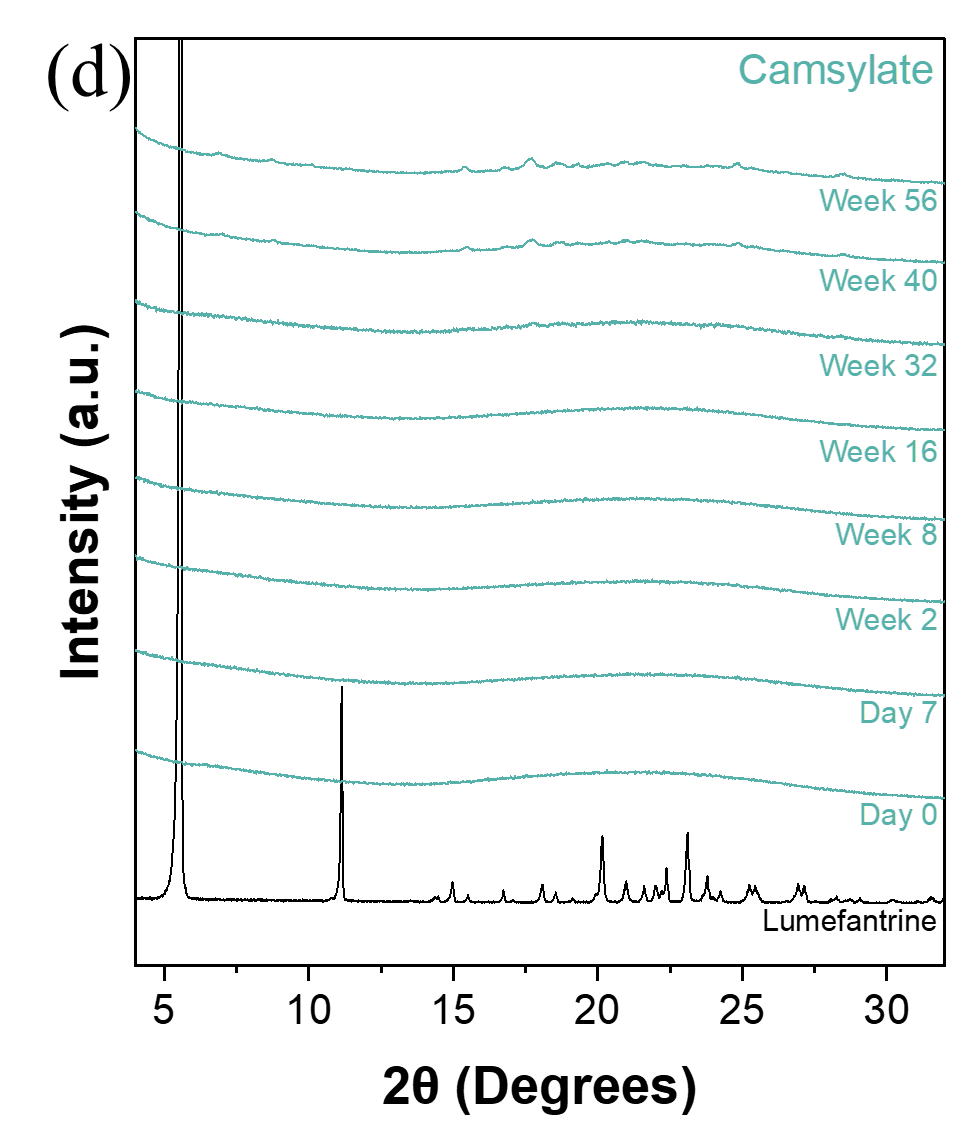 | 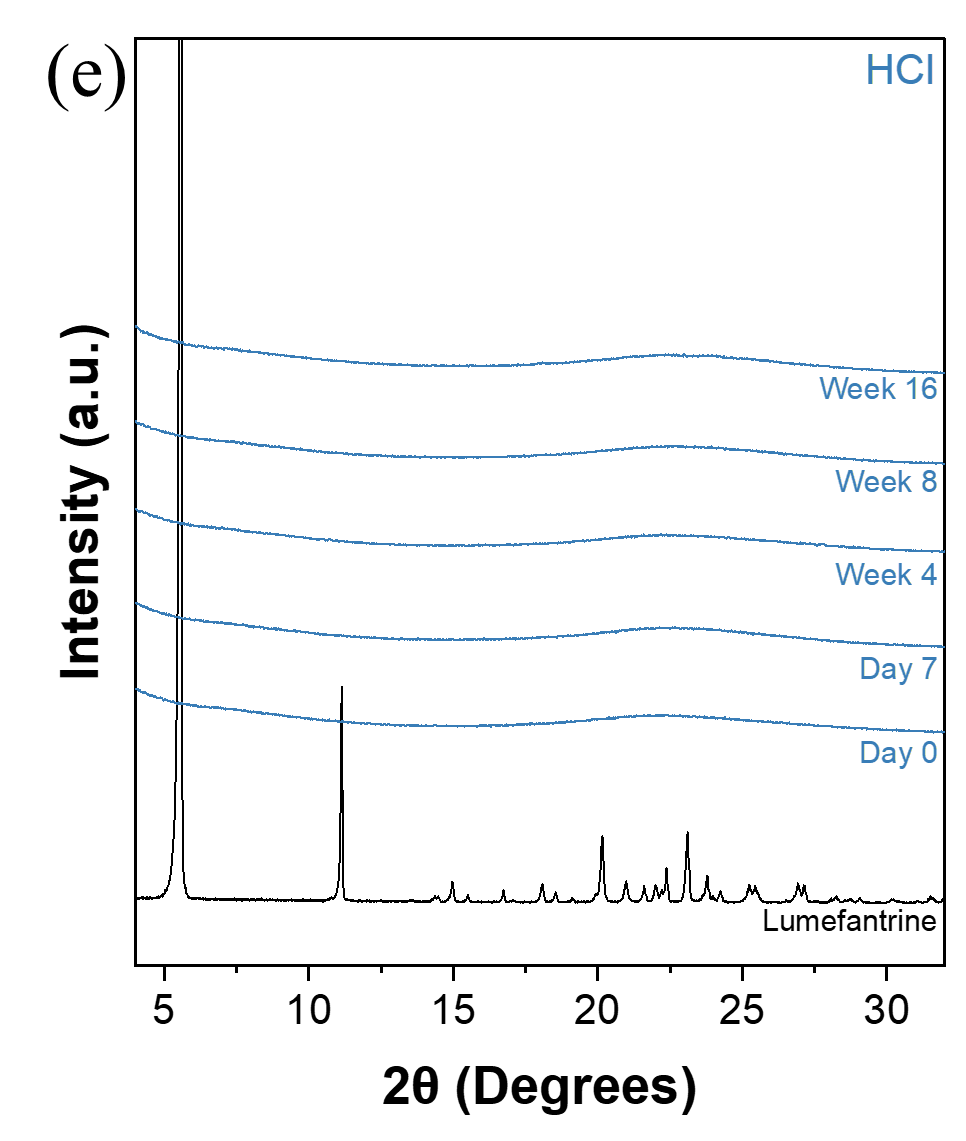 | 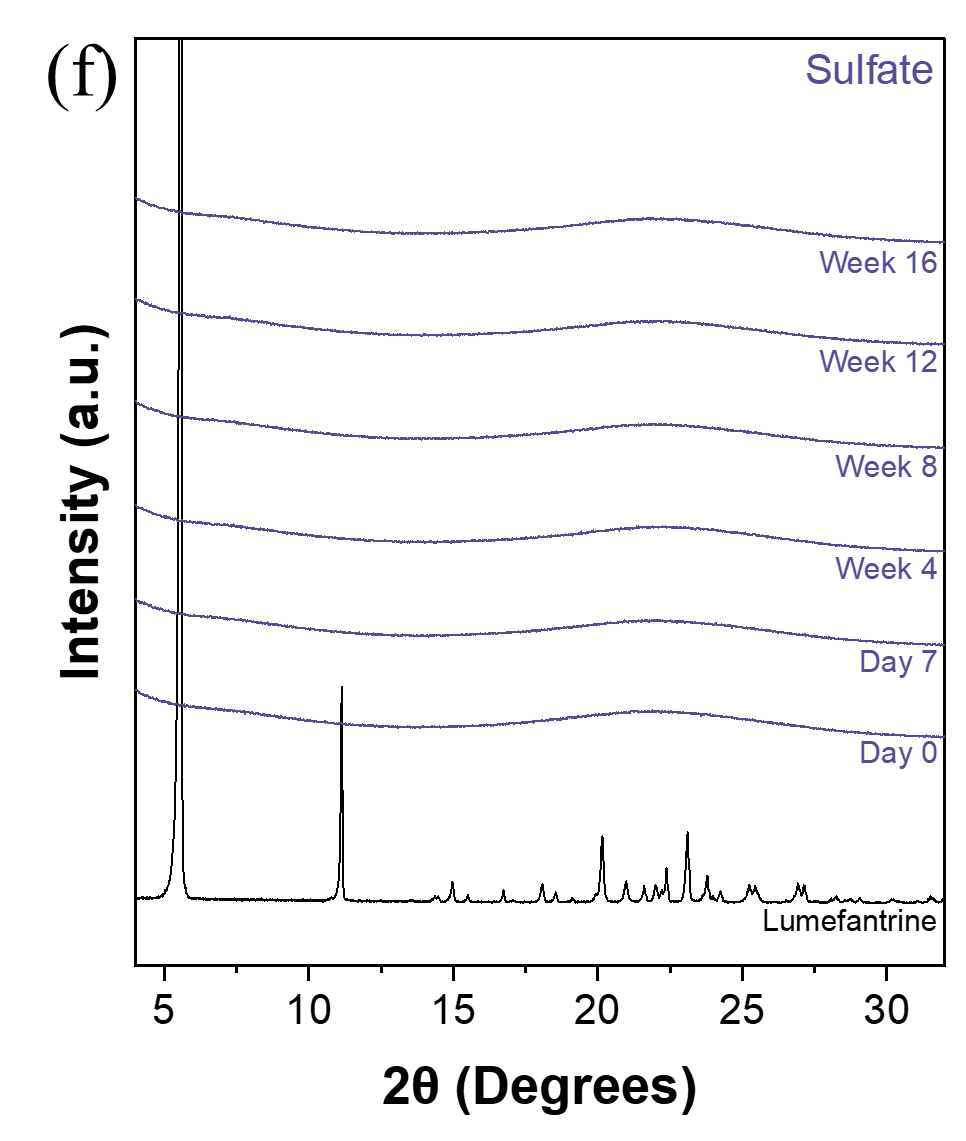 |

Figure S1. PXRD patterns of lumefantrine (a) benzoate, (b) PEG 250 diacid (2:1), (c) besylate, (d) camsylate, (e) HCl, and (f) sulfate salts following open dish storage at accelerated stability conditions of 40°C/75% RH.

## Physical Stability of Lumefantrine Amorphous Solid Dispersions (ASDs)

| 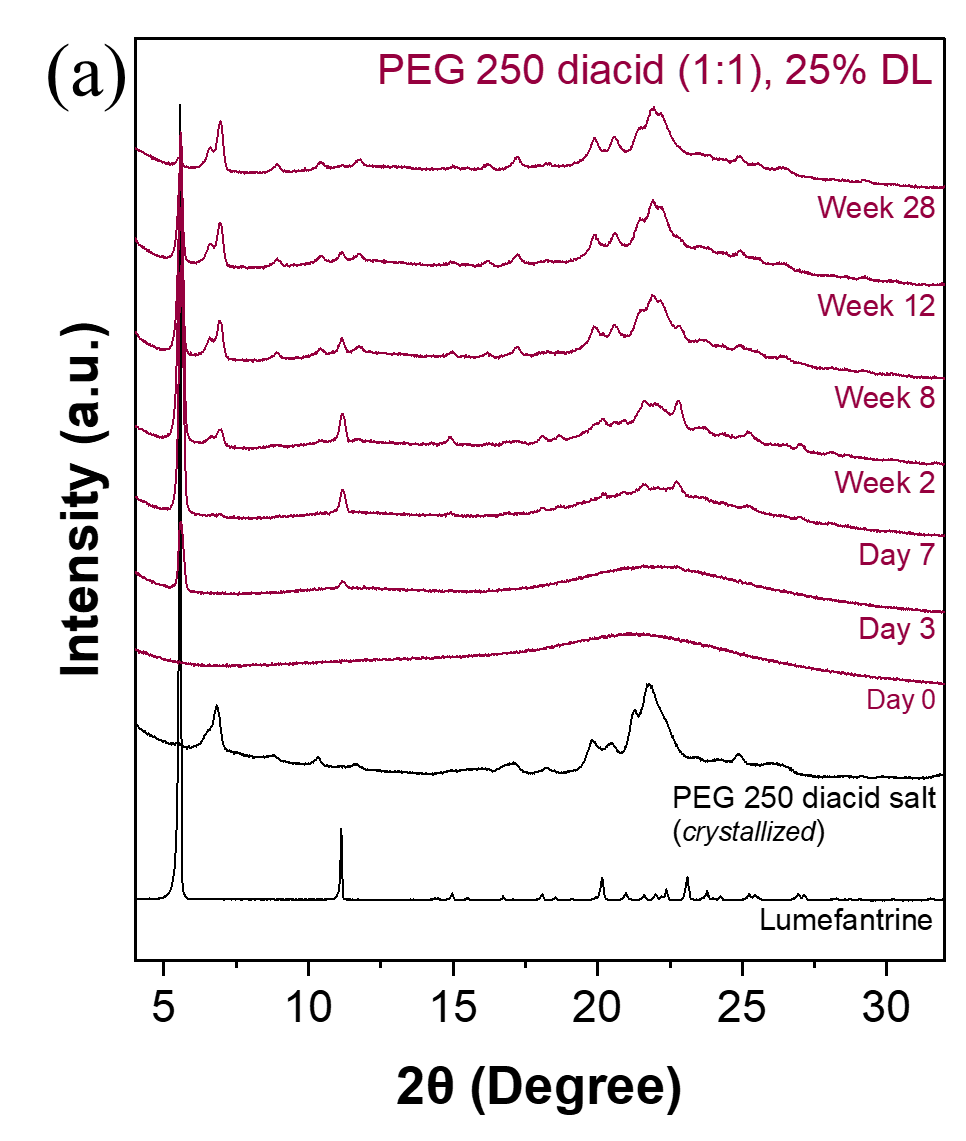 | 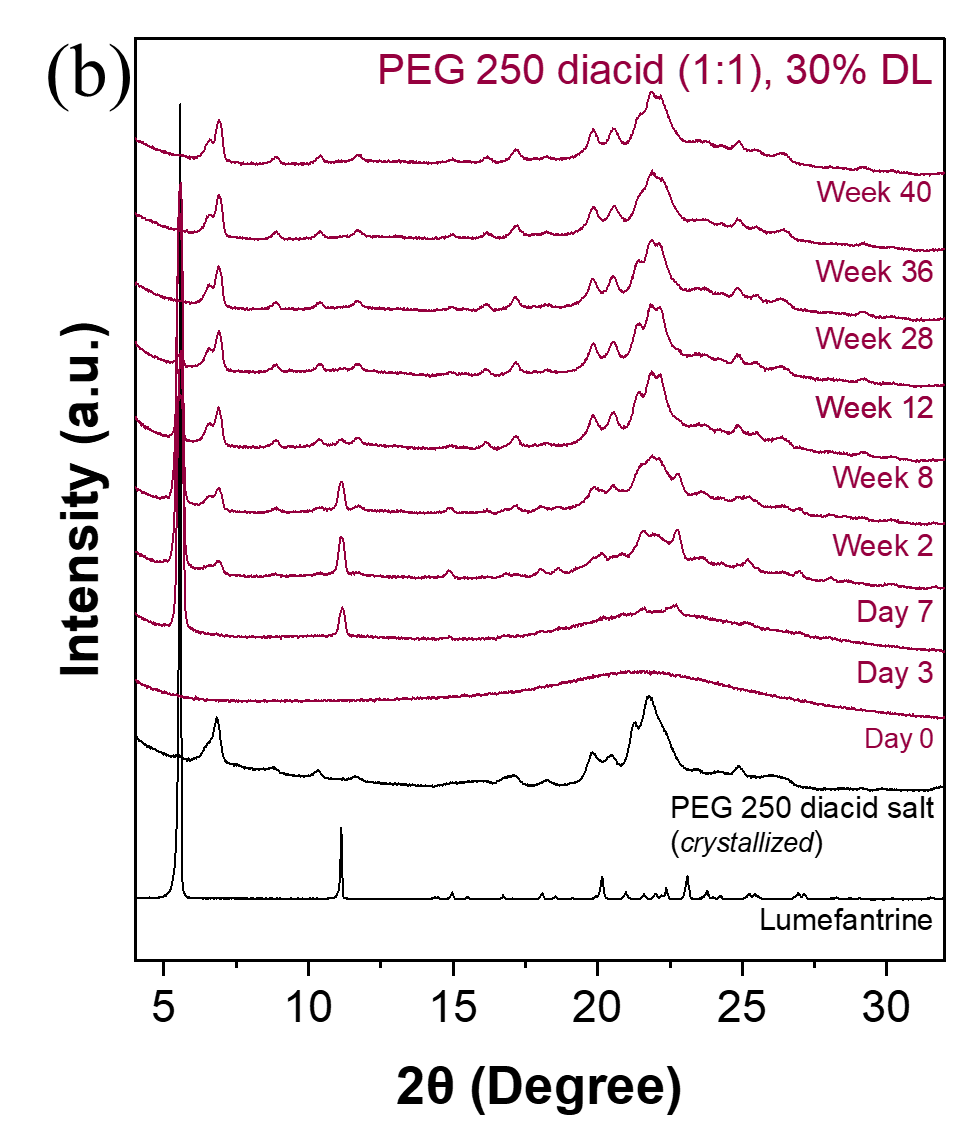 | 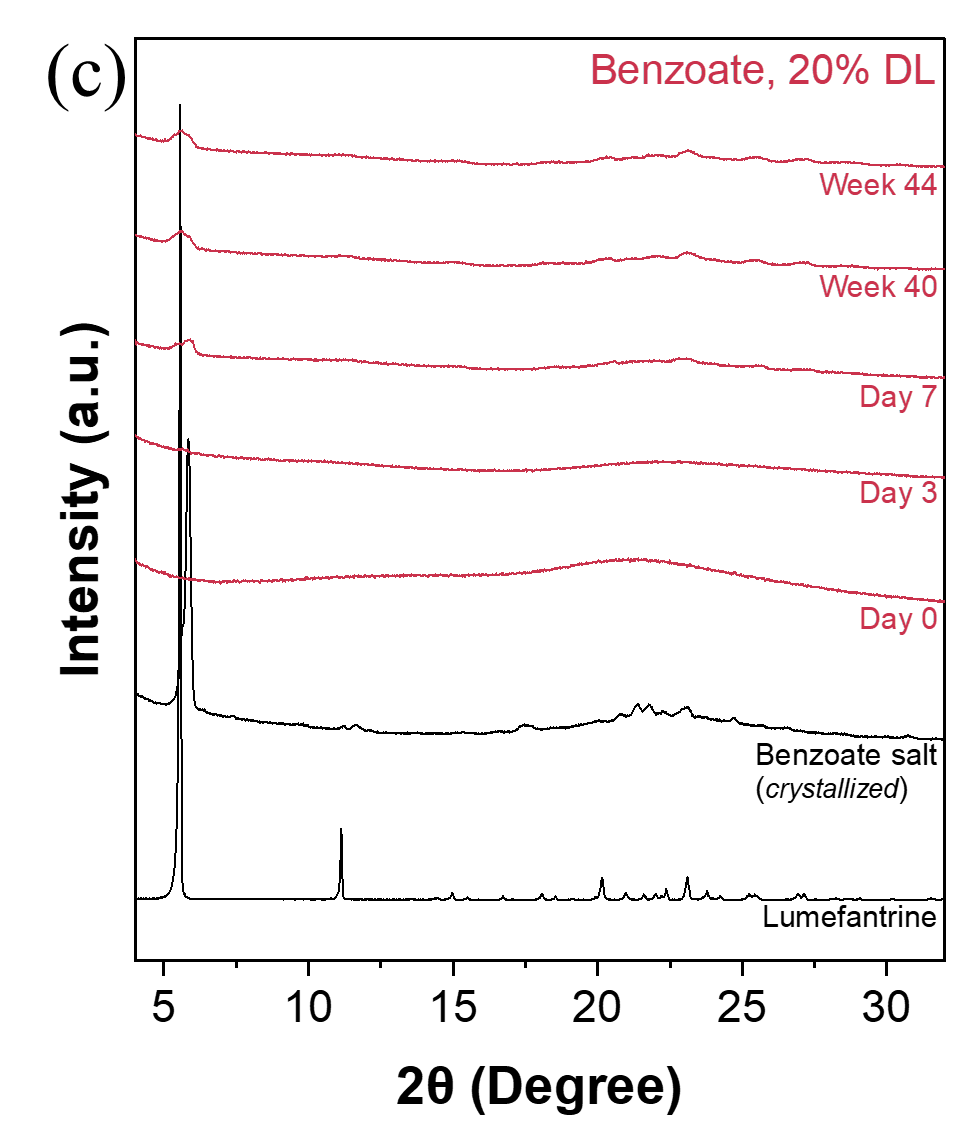 |
| --- | --- | --- |
| 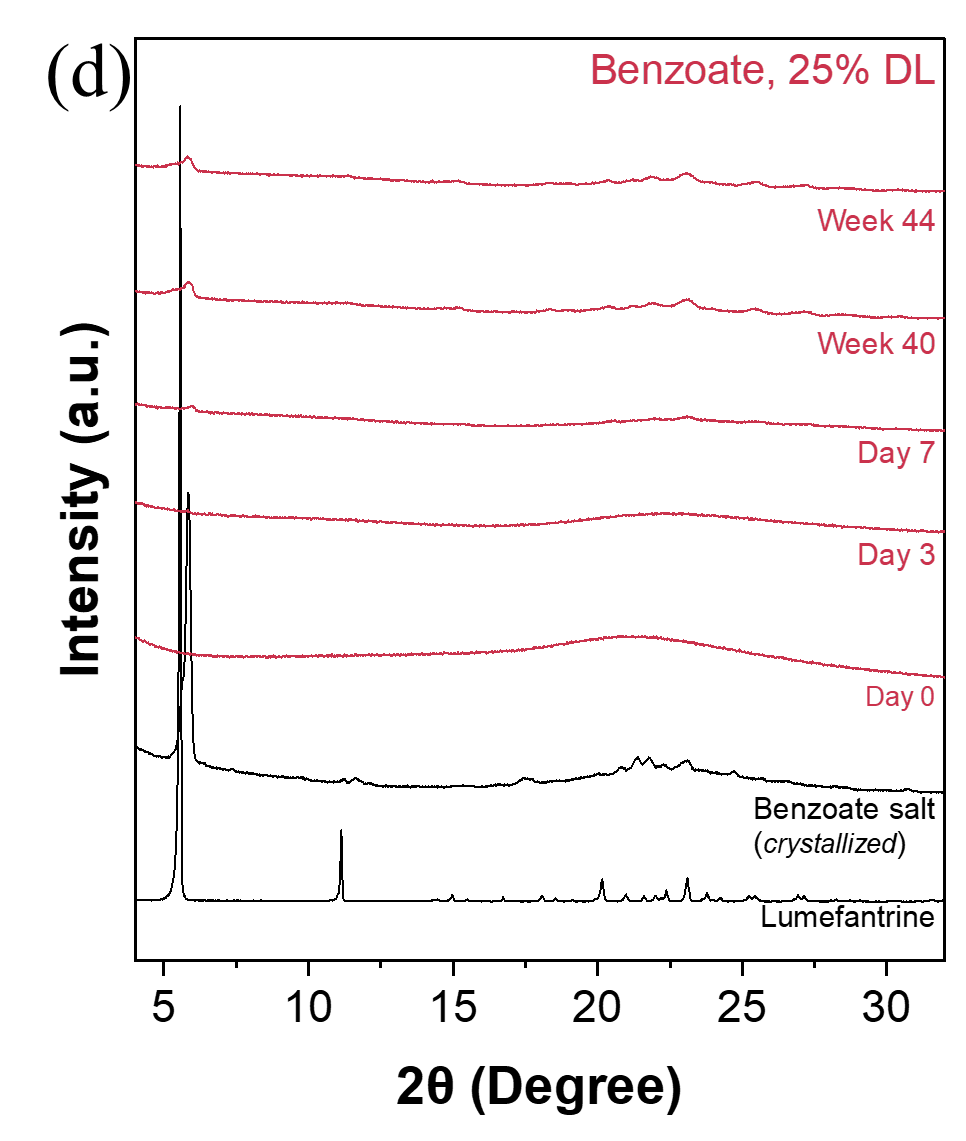 | 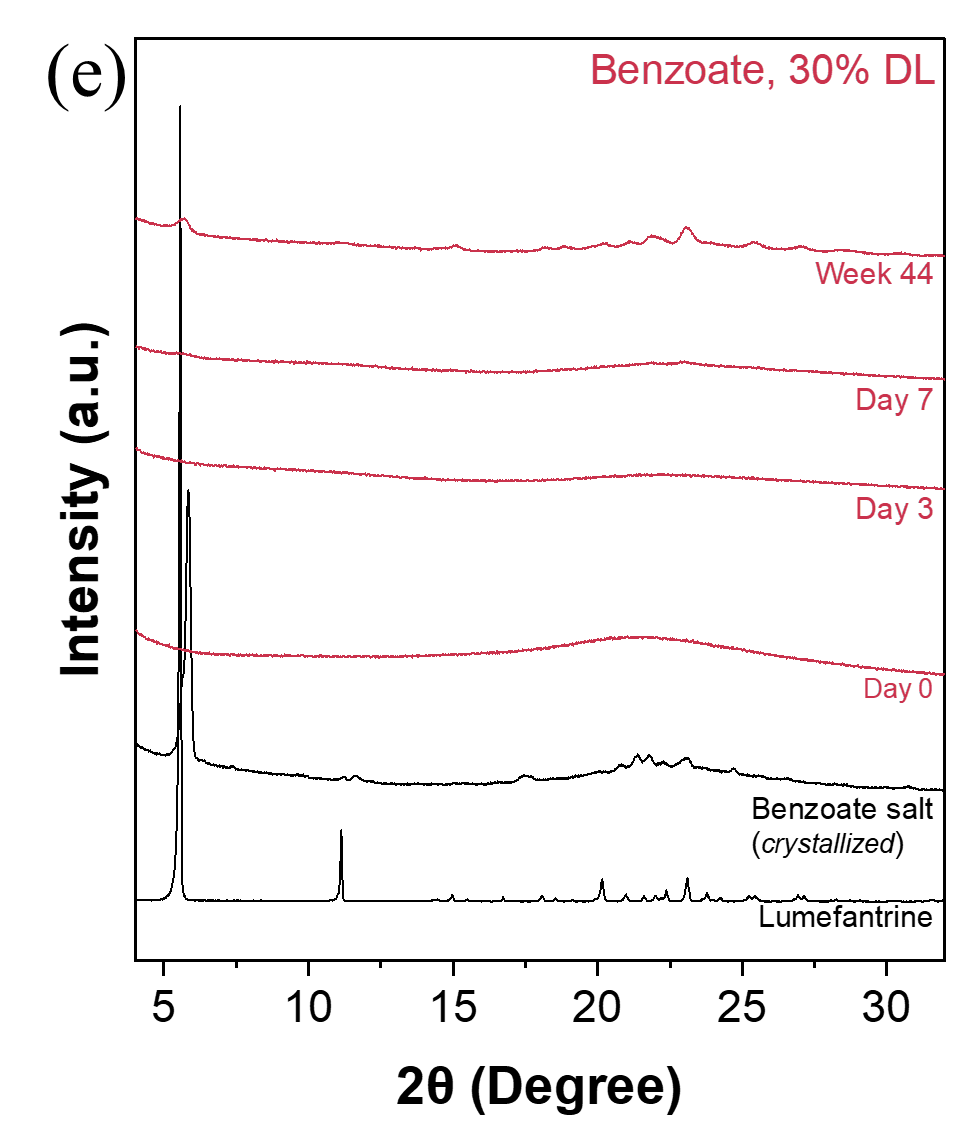 | 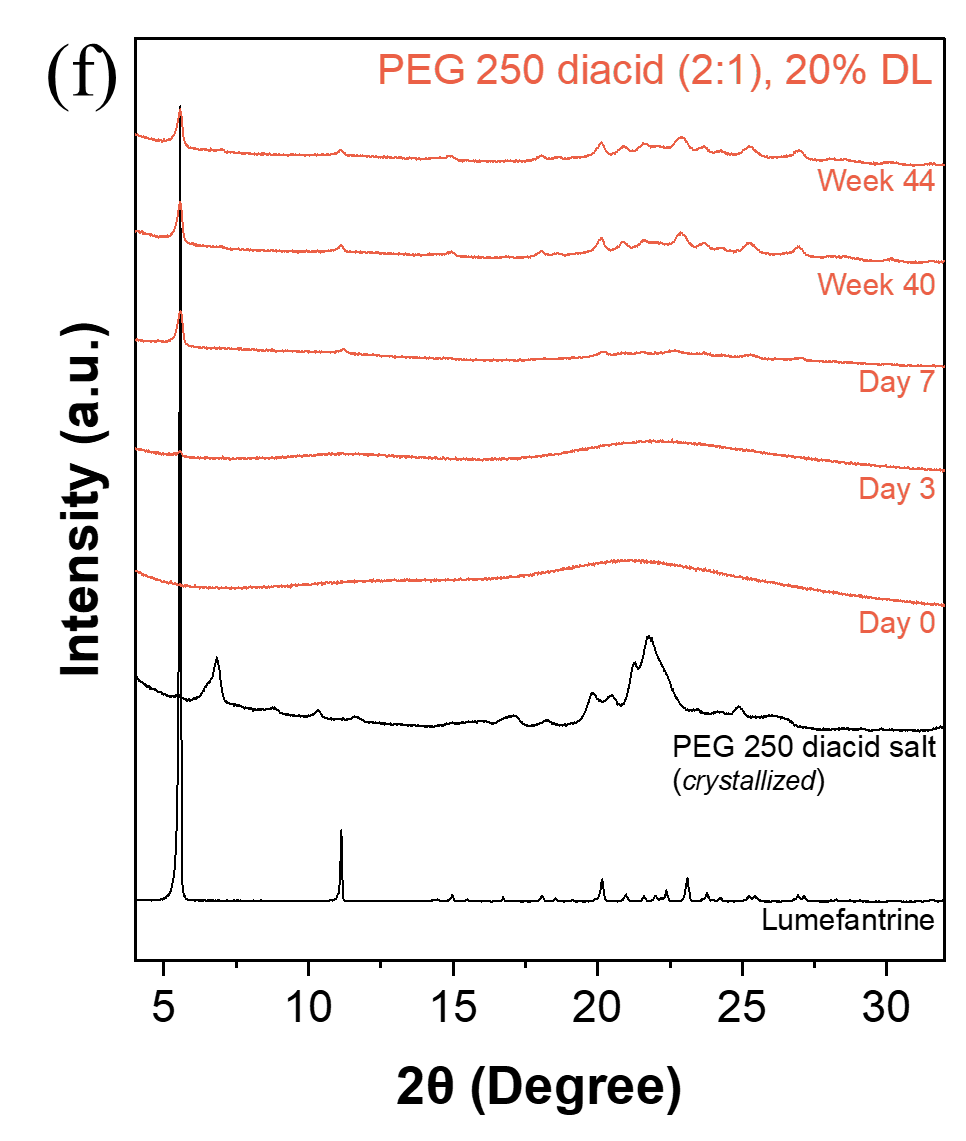 |
| 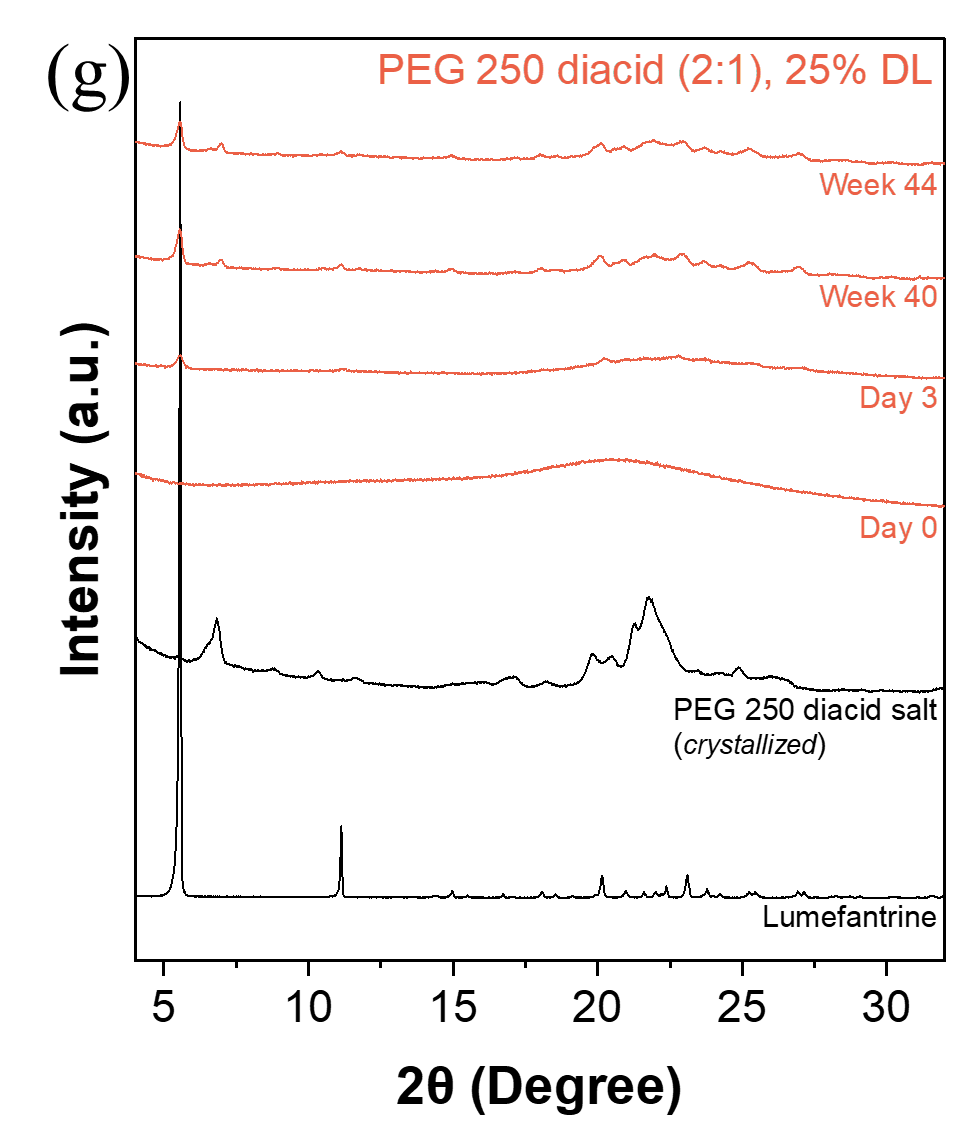 | 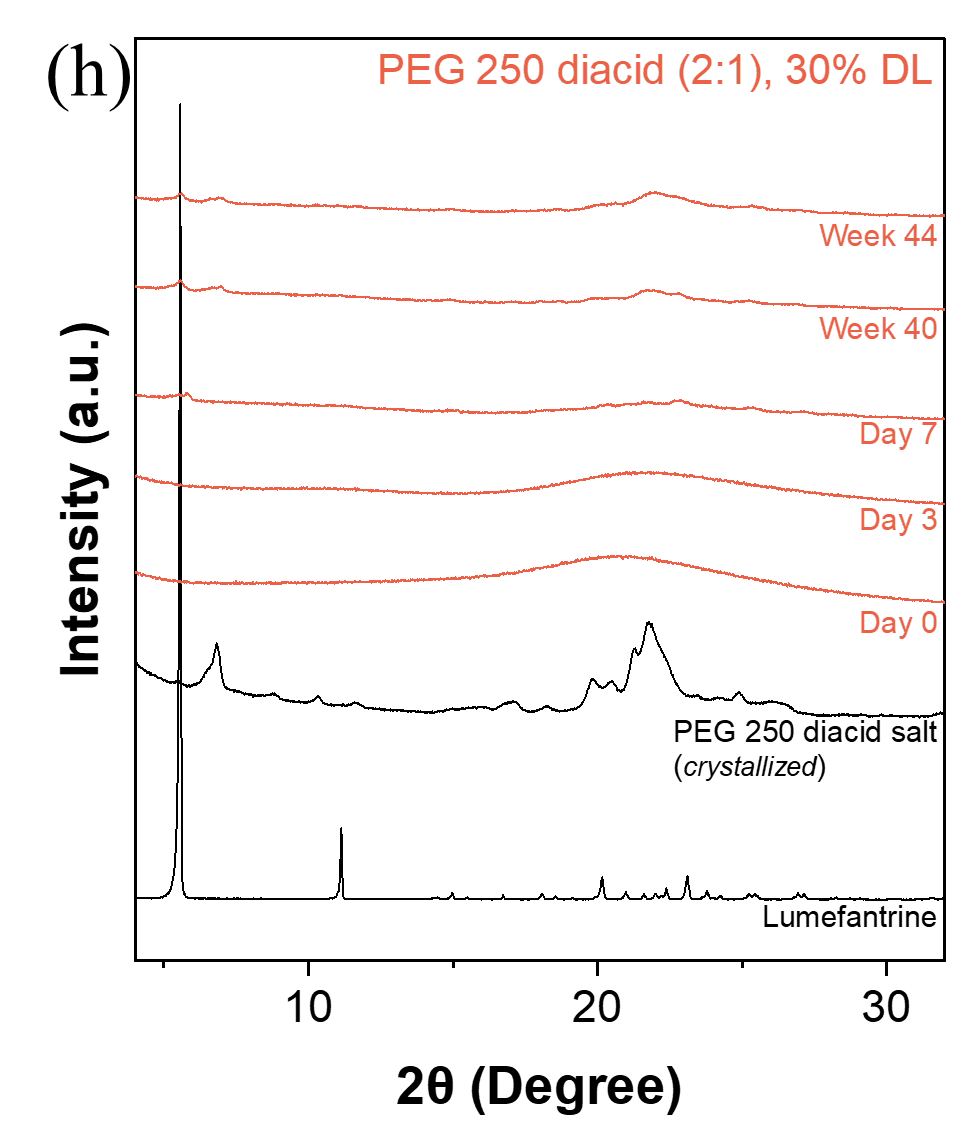 | 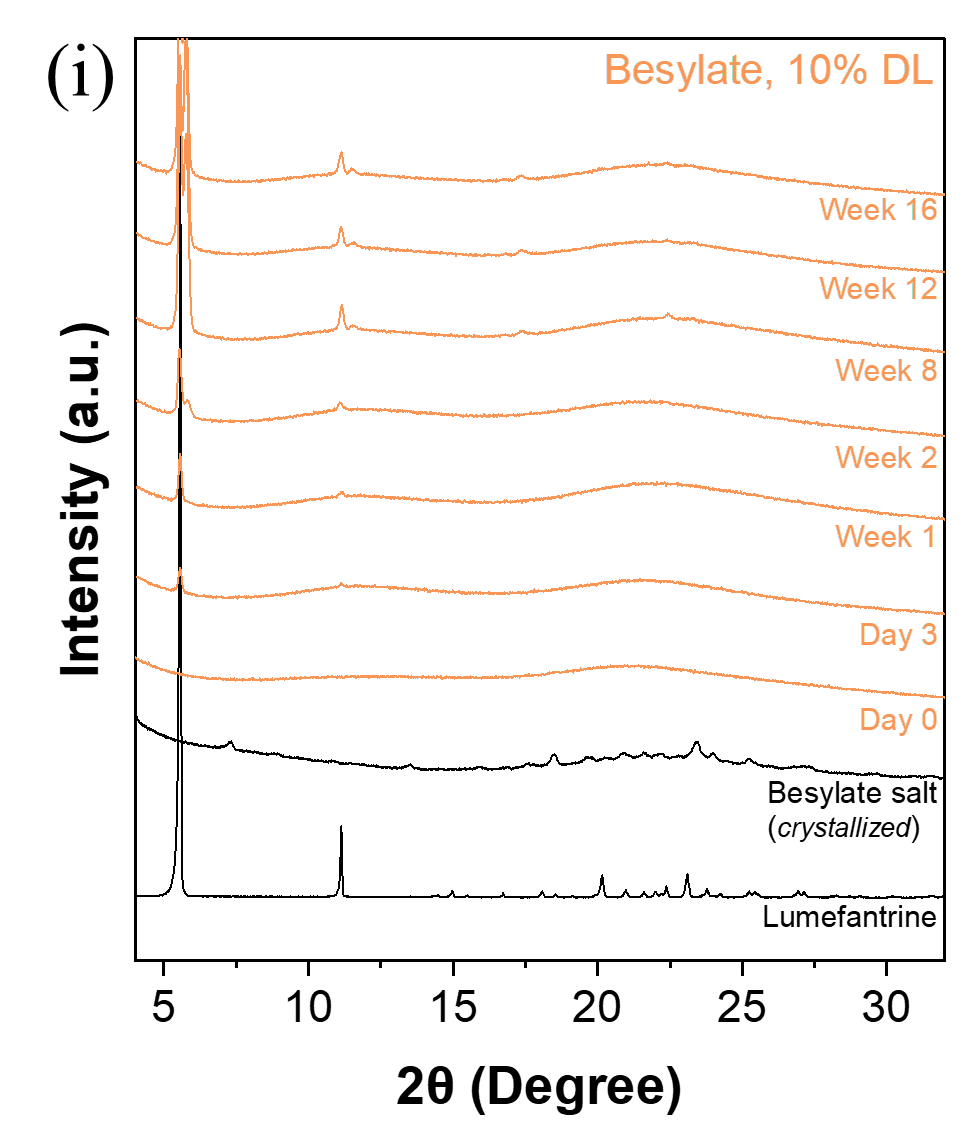 |
| 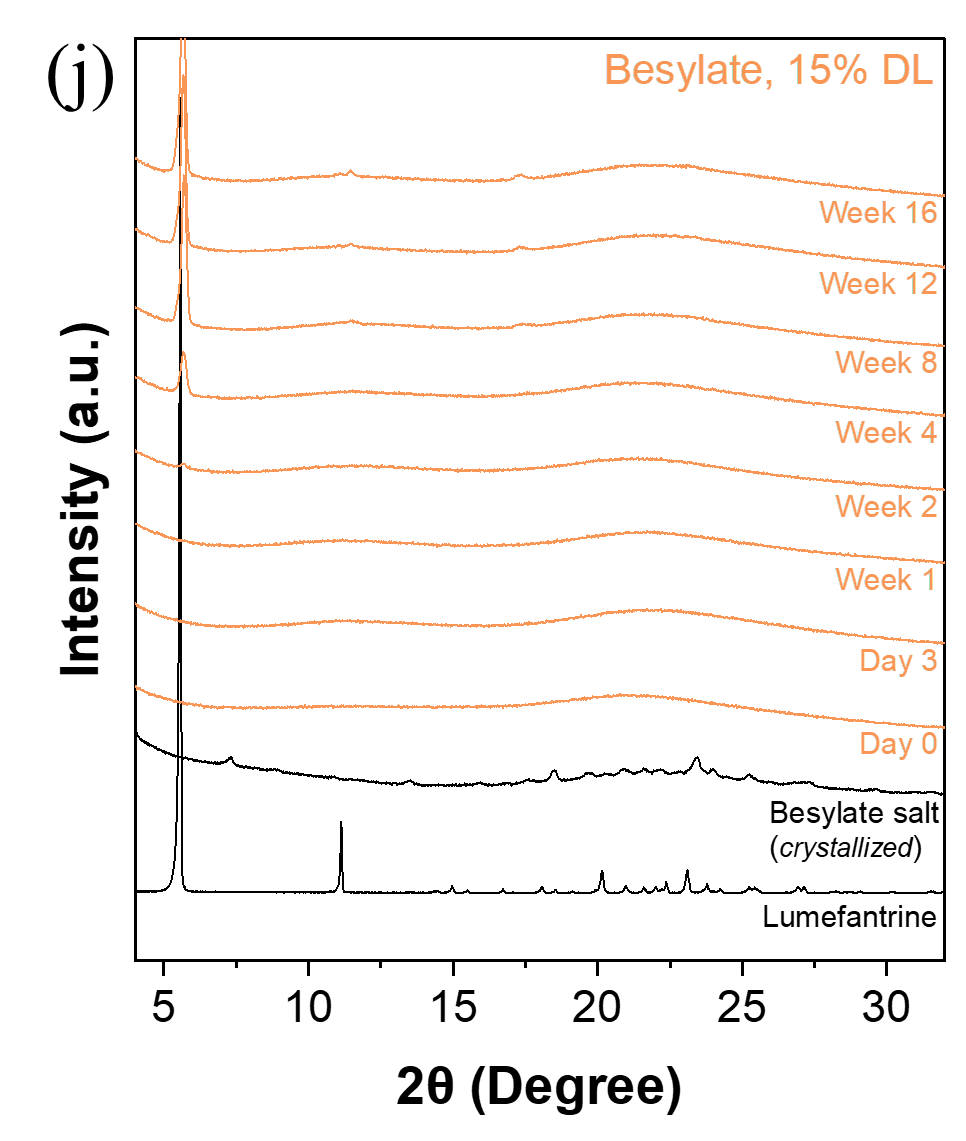 | 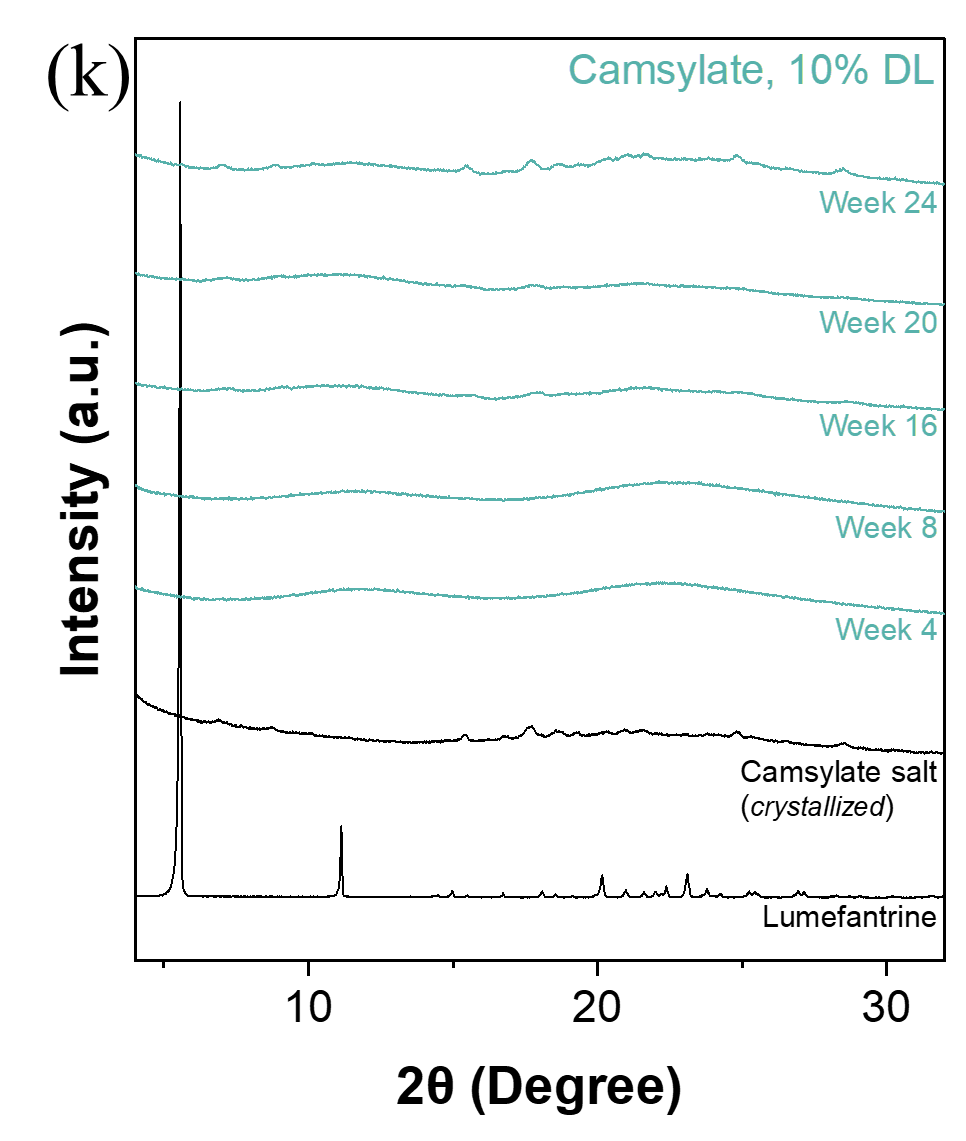 | 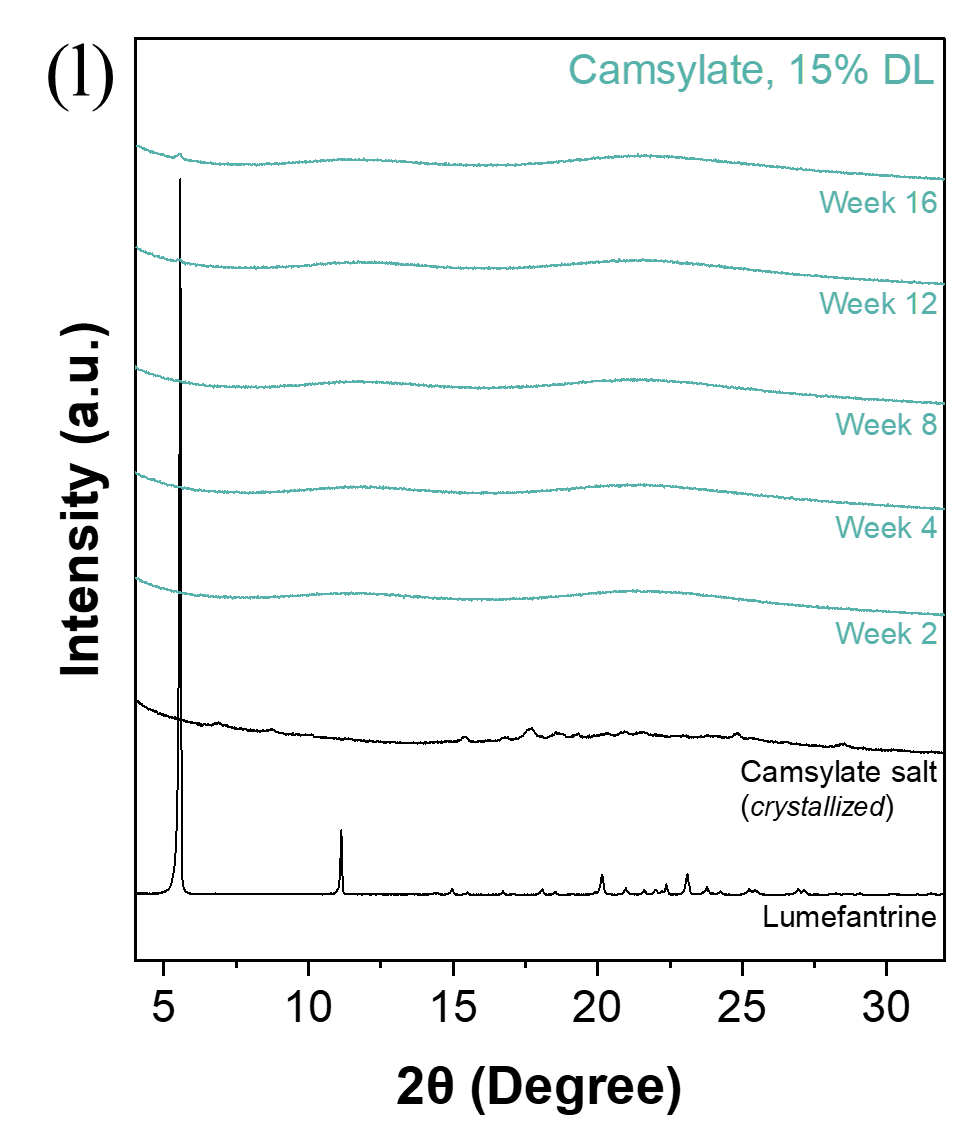 |
| 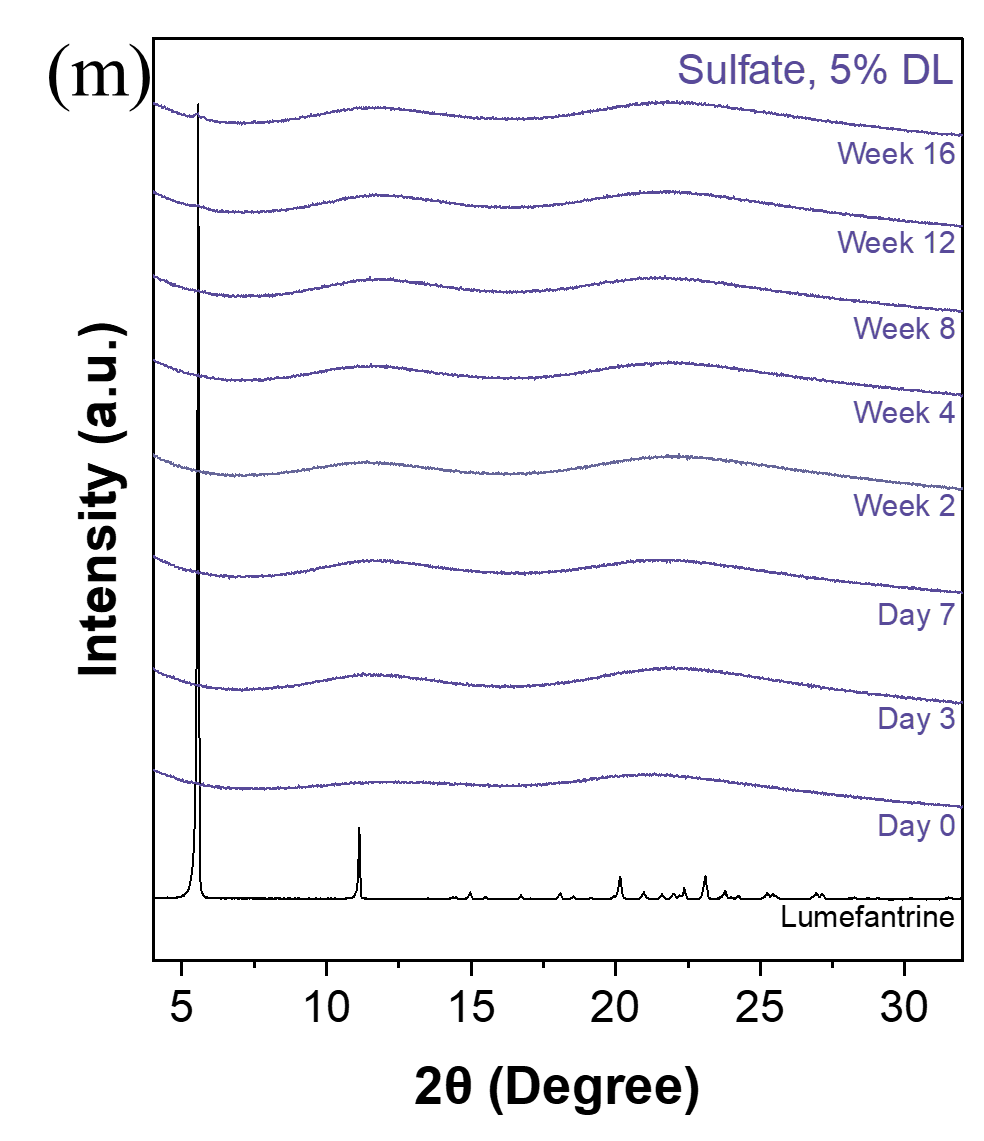 |  |  |

Figure S2. PXRD patterns of lumefantrine PEG 250 diacid–PVPVA (a) 25% DL, (b) 30% DL formulations (1:1), lumefantrine benzoate–PVPVA (c) 20% DL, (d) 25% DL, (e) 30% DL formulations, lumefantrine PEG 250 diacid–PVPVA (f) 20% DL, (h) 25% DL, (h) 30% DL formulations (2:1), lumefantrine besylate–PVPVA (i) 10% DL, (j) 15% DL formulations, lumefantrine camsylate–PVPVA (k) 10% DL, (l) 15% DL formulations, and (m) lumefantrine sulfate–PVPVA 5% DL ASD formulations following open dish storage accelerated stability conditions of 40°C/75% RH.

## Surface Normalized Dissolution of Lumefantrine ASDs

| 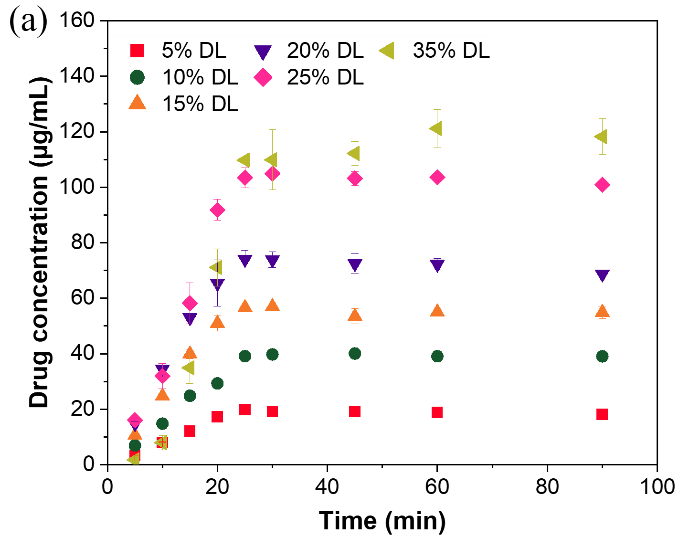 | 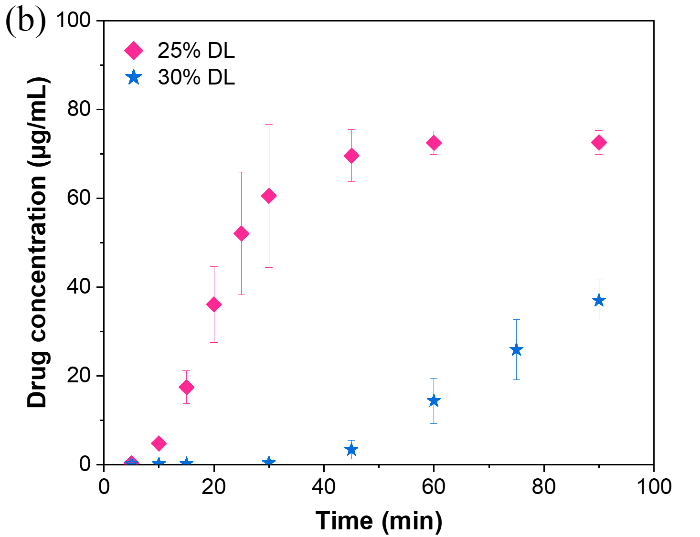 |
| --- | --- |
| 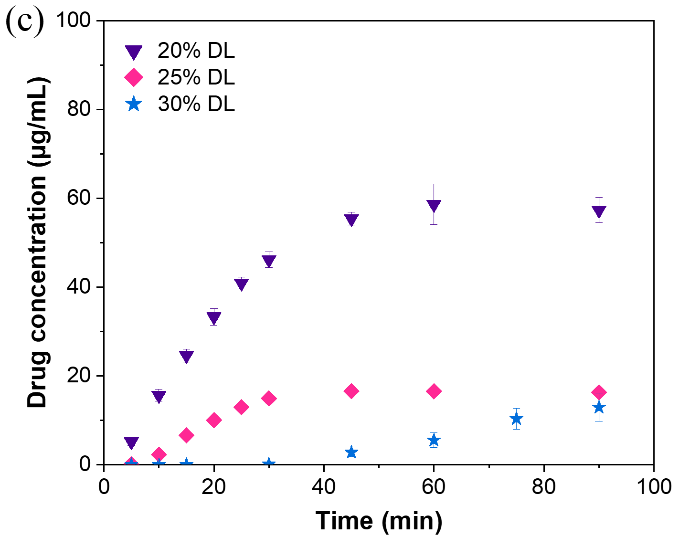 | 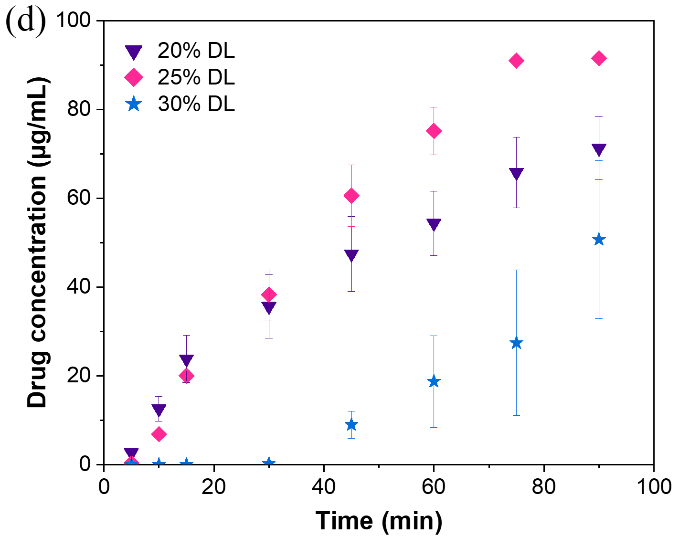 |
| 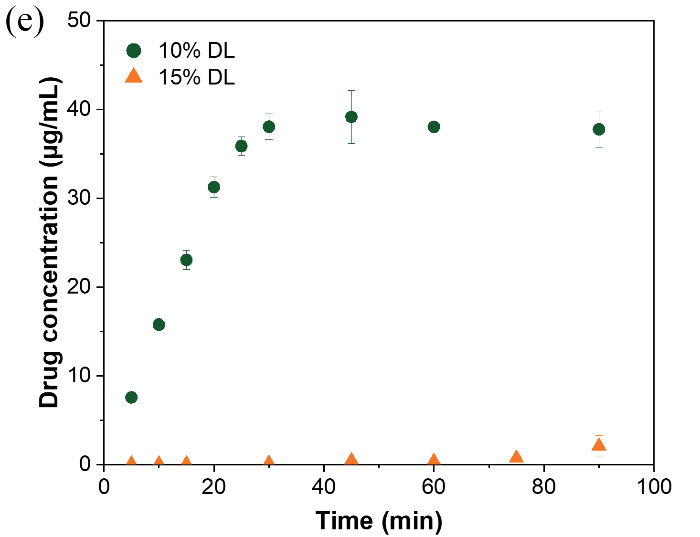 | 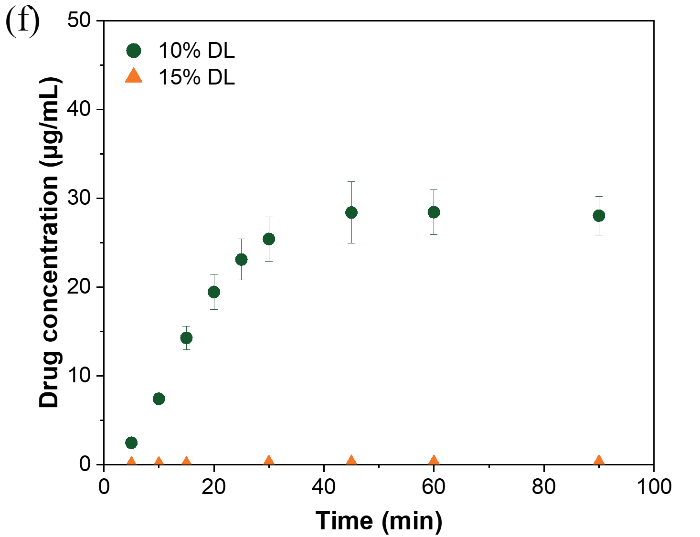 |
| 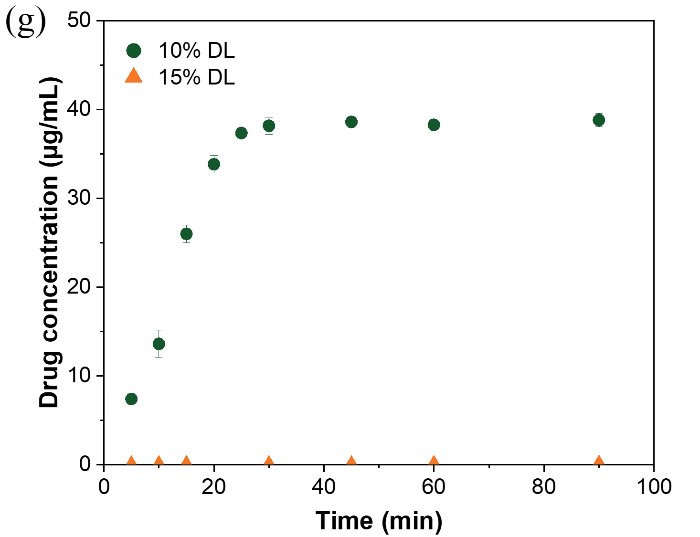 | 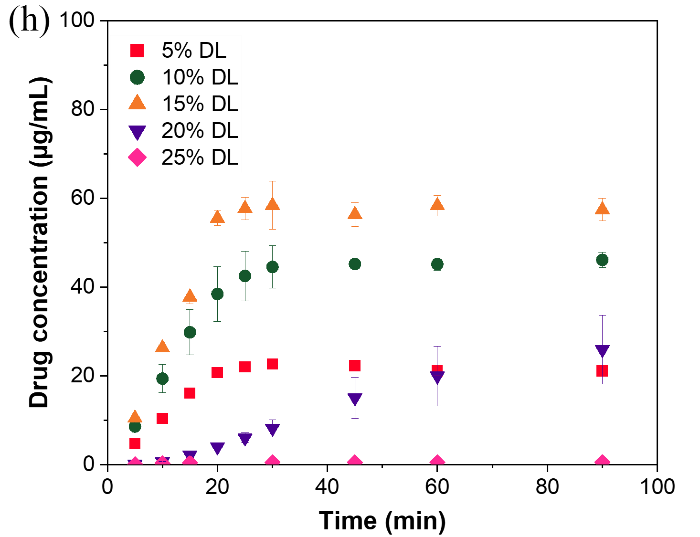 |
| 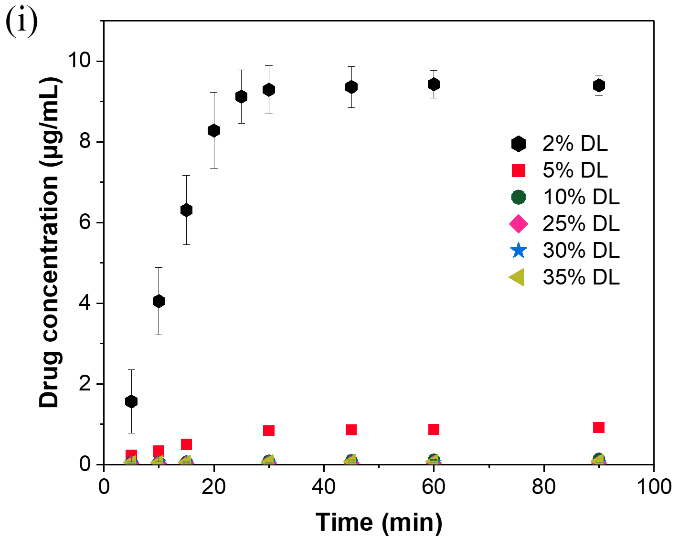 |  |

Figure S3. Drug release profiles of PVPVA-based lumefantrine ASDs prepared with (a) lumefantrine free base, (b) lumefantrine PEG 250 diacid (2:1), (c) lumefantrine benzoate, (d) lumefantrine PEG 250 diacid (1:1), (e) lumefantrine besylate, (f) lumefantrine tosylate, (g) lumefantrine camsylate, (h) lumefantrine HCl, and (i) lumefantrine sulfate. Error bars represent one standard deviation, where *n* = 3.

## Weight Change of Lumefantrine Salt ASDs

Table S1. Percent weight change of select lumefantrine salt ASDs when RH was increased from 0 to 95% ^a^.

| ASD sample | Weight (% change) | | | | | | |
| --- | --- | --- | --- | --- | --- | --- | --- |
|  | 5% DL | 10% DL | 15% DL | 20% DL | 25% DL | 30% DL | 35% DL |
| Lumefantrine benzoate | ^b^ | ^b^ | ^b^ | ^c^ | 40.5 (1.5) | 34.5 (1.2) | ^b^ |
| Lumefantrine PEG 250 diacid (2:1) | ^b^ | ^b^ | ^b^ | 45.6 (0.3) | 46.6 (4.0) | 38.2 (2.5) | ^b^ |
| PEG 250 diacid (1:1) | ^b^ | ^b^ | ^b^ | ^b^ | 38.4 (2.4) | 37.1 (2.0) | ^b^ |
| Lumefantrine besylate | ^b^ | 48.5 (1.2) | 48.4 (1.3) | ^b^ | ^b^ | ^b^ | ^b^ |
| Lumefantrine camsylate | ^b^ | 51.0 (2.0) | 47.2 (1.0) | ^b^ | ^b^ | ^b^ | ^b^ |
| Lumefantrine sulfate | 54.7 (2.0) | 50.4 (0.4) | ^b^ | ^b^ | ^c^ | ^c^ | ^c^ |

^a^Standard errors of the mean are shown in parentheses, where *n* = 2.

^b^ASD not prepared.

^c^Water vapor sorption of ASD not evaluated.

## FTIR Analysis

To further investigate the presence of hydrogen bond interactions between lumefantrine salts and PVPVA, a comparison of the peak heights at 1736 and 1684 cm^−1^ was made. For reference, the peak ratios for neat PVPVA where there is no hydrogen bonding, as well as a strongly interacting system, phenolphthalein (PHPH)–PVPVA ASD at 30% DL [1] were included for comparison. The premise of the comparison is that hydrogen bonding between the drug and polymer will reduce the intensity of the vinylpyrrolidone carbonyl peak at 1684 cm^−1^, relative to the peak at 1736 cm^−1^. As shown in Figure S4, the reference system (PHPH–PVPVA), which is known to have drug–polymer hydrogen bonding interactions, had the highest peak ratio. The PEG 250 diacid at a 1:1 ratio, where there is an unreacted COOH group, as well as the tosylate salt, showed the highest extent of interaction with the polymer out of the salts. Less interaction was seen with the PEG 250 diacid (2:1) salt, while the free base, benzoate, camsylate, HCl, and sulfate salts showed minimal interaction.

 Figure S4. Peak height ratios between the peaks at 1736 and 1684 cm^−1^ for various lumefantrine ASDs at 30% DL. An increase in the peak height ratio suggests the presence of drug–polymer hydrogen bond interactions.

*Spectral subtraction was performed to eliminate the influence of camphorsulfonic acid on the peak height ratio. Details on spectral subtraction is presented below.

## Subtraction Spectroscopy

Subtraction spectroscopy was used to eliminate the influence of camphorsulfonic acid on the peak height ratio. The spectra of lumefantrine camsylate–PVPVA 30% DL ASD before and after spectral subtraction are shown in Figure S5. Spectral subtraction was performed using the GRAMS/AI™ Spectroscopy Software (Version 9.2, Thermo Fisher Scientific, Waltham, MA). Briefly, the software subtracts two files using the following equation:

| Result file = Sample file − (Subtrahend file × Subtraction factor) | **Eq. S1** |
| --- | --- |

The sample file was the spectrum of lumefantrine camsylate–PVPVA 30% DL ASD, while the subtrahend file was the spectrum of lumefantrine camsylate salt. Iteration was performed until the change in the subtraction factor value was less than 0.01%.

**Figure S5. The spectra of lumefantrine camsylate–PVPVA 30% DL ASD before (black) and after (red) spectral subtraction.**

## Confocal Microscopy


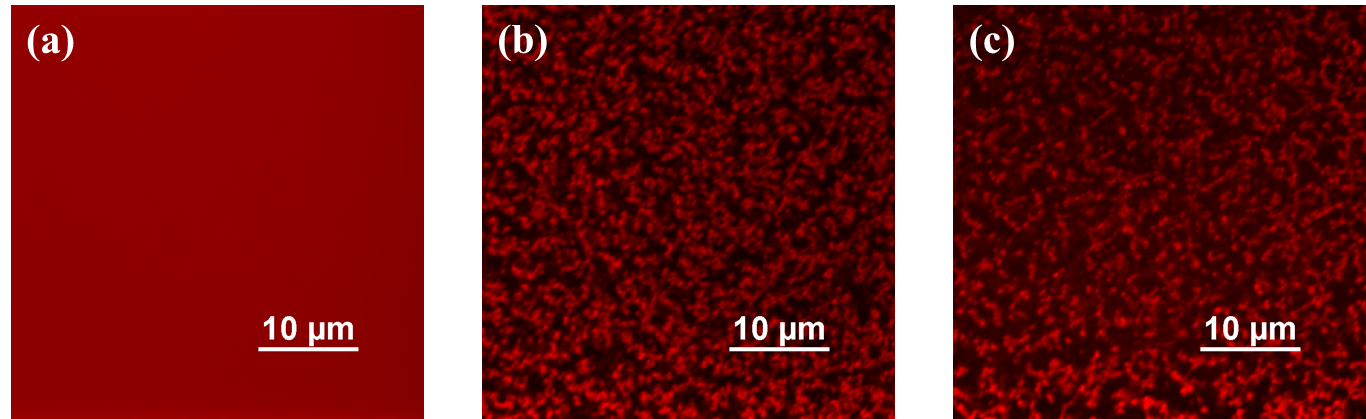


Figure S6. Lumefantrine–PVPVA 5% DL ASD film (a) 0 min, (b) 10 min, and (c) 30 min after exposure to water vapor in an *in situ* humidity chamber showing tendency to undergo amorphous–amorphous phase separation.


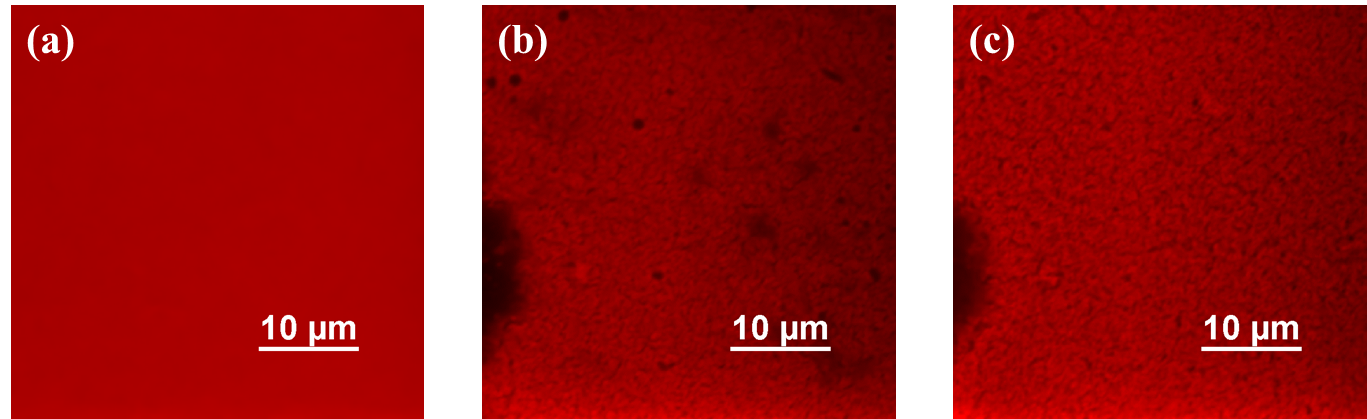


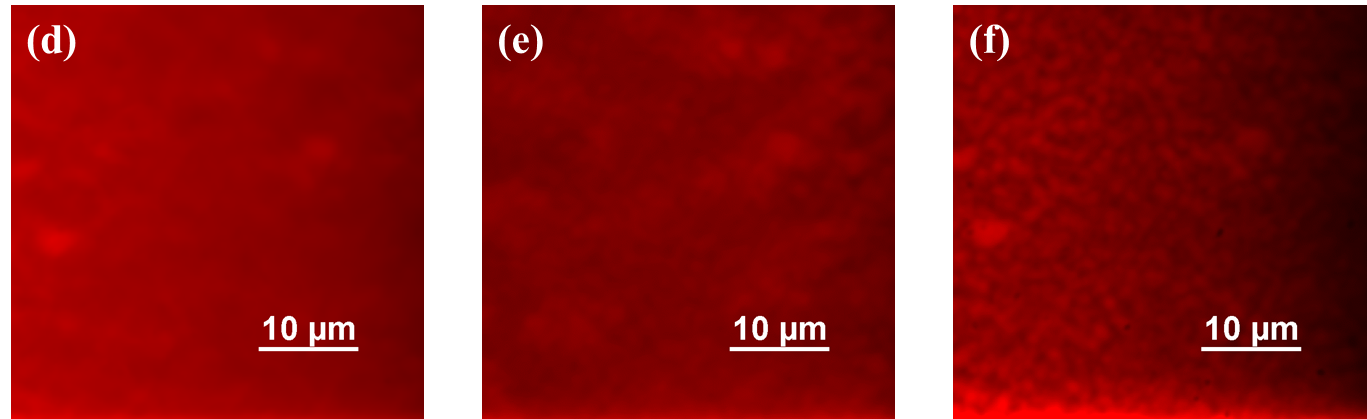


Figure S7. Lumefantrine benzoate–PVPVA 20% DL ASD film (a) 0, (b) 10 min, and (c) 30 min and lumefantrine benzoate–PVPVA 30% DL ASD film (d) 0 min, (e) 10 min, and (f) 30 min after exposure to water vapor in an *in situ* humidity chamber showing tendency to undergo amorphous–amorphous phase separation.

**
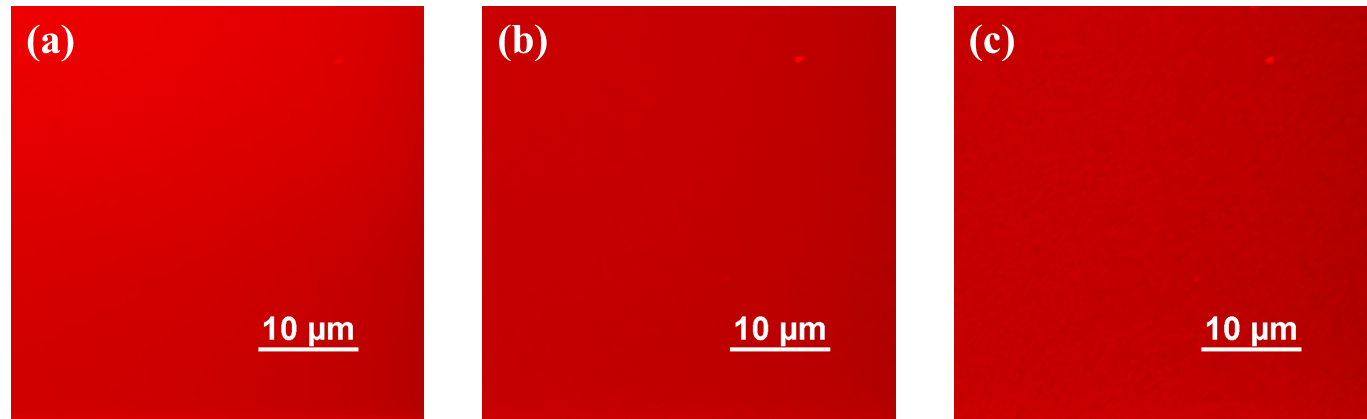
**

Figure S8. Lumefantrine sulfate–PVPVA 5% DL ASD film (a) 0 min, (b) 10 min, and (c) 30 min after exposure to water vapor in an *in situ* humidity chamber showing tendency to undergo amorphous–amorphous phase separation.

## Reference

[1] C. Que, A. Deac, D.Y. Zemlyanov, Q. Qi, A.S. Indulkar, Y. Gao, G.G.Z. Zhang, L.S. Taylor, Impact of drug–polymer intermolecular interactions on dissolution performance of copovidone-based amorphous solid dispersions, Mol. Pharm., 18 (2021) 3496-3508, https://doi.org/10.1021/acs.molpharmaceut.1c00419.
